# Supplementary material for: Inferring assembly-curving trends of bacterial micro-compartment shell hexamers from crystal structure arrangements
Source: PLoS Comput Biol. 2023 Apr 5;19(4):e1011038. doi: 10.1371/journal.pcbi.1011038 (PMC10109471; doi:10.1371/journal.pcbi.1011038)
Supplement: S1 Table — Data were compiled from references indicated in the last column. Objects were imaged by TEM directly after protein overexpression inside living cells (generally E. coli), or by TEM, cryo-EM or AFM with purified proteins (in vitro). Curved-implying objects are highlighted with blue letters, black for flat structures. NA: not applicable. (DOCX) [file pcbi.1011038.s001.docx]

**Table S1. Nano-assemblies characterized for individual BMC-H.** Data were compiled from references indicated in the last column. Objects were imaged by TEM directly after protein overexpression inside living cells (generally *E. coli*), or by TEM, cryo-EM or AFM with purified proteins (*in vitro*). Curved-implying objects are highlighted with blue letters, black for flat structures. NA: not applicable.

| **BMC-H** | **Species** | **Method** | **Assemblies**  **in cells** | **Assemblies**  ***in vitro*** | **Details** | **Ref.** |
| --- | --- | --- | --- | --- | --- | --- |
| **CcmK1** | Hal. sp. Pcc7418 | TEM | None visible | Not done |  | 1 |
| **CcmK1** | Syn. sp. PCC 6803 | AFM | Not applicable (NA) | **2D assemblies** (cup-like patches) |  | 2 |
| **CcmK1** | Syn. sp. PCC 6803 | TEM | Not done | **2D-crystal assemblies** | lipid-air interface | 3 |
| **CcmK2** | Hal. sp. Pcc7418 | TEM | None visible | Not done |  | 1 |
| **CcmK2** | Syn. elongatus PCC 7942 | AFM | Not done | **2D assemblies** (occasionally with stripes) |  | 2 |
| **CcmK2** | Syn. elongatus PCC 7942 | TEM | None visible | Not done |  | 4 |
| **CcmK2** | Syn. sp. PCC 6803 | AFM | NA | **2D assemblies** (occasionally with stripes) |  | 2 |
| **CcmK2** | Syn. sp. PCC 6803 | TEM | Not mentioned | **Spheroids (30-50 nm)** |  | 5 |
| **CcmK2** | Syn. sp. PCC 6803 | TEM | Not done | **2D-crystal assemblies** (stacks) | lipid-air interface | 3 |
| **CcmK2** | T. elongatus BP-1 | TEM |  | **bodies** (100-300 nm) | artifact? | 6 |
| **CcmK4** | Syn. elongatus PCC 7942 | TEM | None visible | Not done |  | 4 |
| **CcmK4** | Syn. sp. PCC 6803 | AFM | NA | **2D assemblies** |  | 2 |
| **CcmK4** | Syn. sp. PCC 6803 | TEM | Not done | **2D-crystal assemblies** (stacks) | lipid-air interface | 3 |
| **BMC-H** | Hal. ochraceum | TEM | **Swiss-rolls** |  |  | 7 |
| **BMC-H** | Hal. ochraceum | AFM | NA | **2D flat patches** | Untagged  presence of Mg2+ | 7 |
| **BMC-H** | Hal. ochraceum | TEM | None visible | **2D flat assemblies** |  | 8 |
| **BMC-H** | Hal. ochraceum | TEM | **Swiss-rolls** | Not done |  | 4 |
| **BMC-H** | Hal. ochraceum | TEM | **Swiss-rolls** | Not done |  | 1 |
| **BMC-H** | Hal. ochraceum | TEM | **Swiss-rolls** |  |  | 9 |
| **BMC-H K28A** | Hal. ochraceum | AFM | NA | **2D flat patches**  two-stacked and larger than WT | slower exchange dynamics than WT | 7 |
| **BMC-H K28A** | Hal. ochraceum | TEM | Amorphous open rolls? | Not done |  | 1 |
| **BMC-H K28P** | Hal. ochraceum | TEM | **Nanotubes**  or layered **2D-sheets**? | Not done |  | 1 |
| **BMC-H R78A** | Hal. ochraceum | AFM | NA | **2D flat patches** smaller patches than WT | exchange dynamics  comparable to WT | 7 |
| **BMC-H R78A** | Hal. ochraceum | TEM | Incipient rolls? | Not done |  | 1 |
| **BMC-H** | Hal. ochraceum | X-ray | NA | **icosahedral shell** | artificial fusion of two BMC-H | 10 |
| **EtuA** | Clos.kluyveri | TEM | **Nanotubes** | Not soluble using urea |  | 11 |
| **EutM** | Clos. difficile F. gelatini T. saccharolyt. | TEM | Not mentioned | **Fibres and 2D-assemblies** |  | 12 |
| **EutM** | Clos. difficile | TEM | amorphous **swiss-rolls** or irregular **nanotubes?** |  |  | 13 |
| **EutM** | Sal. enterica | TEM | Thick **filaments** | Not mentioned |  | 14 |
| **EutM** | S. enterica M. hydrocarb. T. linaloolentis C. thermarum D. thermocist. P. hadalis A. metalliredigens F. gelatini | TEM | Not mentioned | rolled-up wide **nanotubes** not completely closed |  | 12 |
| **EutS** | Clos. difficile |  | None visible |  |  | 13 |
| **EutS** | Sal. enterica | TEM | **spheroid/polyhedra?** | Not mentioned |  | 14 |
| **PduA** | Cit. freundii | TEM | **Nanotube** bundles | Not mentioned |  | 15 |
| **PduA** | Cit. freundii | TEM | **Nanotubes** | Not done |  | 1 |
| **PduA** | Sal. enterica | TEM | **Rod-like** objects | Not mentioned | solubilized inclusion bodies | 16 |
| **PduA** | Sal. enterica | TEM | **Nanotubes** | Not done |  | 4 |
| **PduA** | Cit. freundii | TEM | Not mentioned | **Nanotubes (20 nm)** |  | 17 |
| **PduA** | Sal. enterica | TEM | **Nanotube** bundles | Not mentioned |  | 18 |
| **PduA*** | Cit. freundii | TEM | **Filaments/nanotubes** | Not mentioned | *Artificial extension | 15 |
| **PduA*** | Cit. freundii | TEM | **Nanotube** bundles | Not mentioned | *Artificial extension | 19 |
| **PduA*** | Cit. freundii | TEM | **Nanotube** bundles | Not mentioned | *C-ter modified | 20 |
| **PduA’** | Sal. enterica | X-ray | NA | **Dodecahedral cage** | ‘ Circular permutation Organized as pentamer | 21 |
| **PduA K26A** | Sal. enterica | TEM | None visible | Not mentioned |  | 18 |
| **PduA* K26A** | Cit. freundii | TEM | layered **2D sheets** | Not mentioned | *Artificial extension | 19 |
| **PduA* K26D** | Cit. freundii | TEM | None visible | Not mentioned | *Artificial extension | 19 |
| **PduA* R79A** | Cit. freundii | TEM | layered **2D sheets** | Not mentioned | *Artificial extension | 19 |
| **PduA* V51A** | Cit. freundii | TEM | **Nanotube** bundles | Not mentioned | *Artificial extension | 19 |
| **PduA* V51D** | Cit. freundii | TEM | None visible | Not mentioned | *Artificial extension | 19 |
| **PduJ** | Sal. enterica | TEM | **Nanotube** bundles | Not mentioned |  | 18 |
| **PduJ K25A** | Sal. enterica | TEM | None visible | Not mentioned |  | 18 |
| **RmmH** | Myc. smegmatis | TEM | **Nanotubes** | **nanotubes** | Triton X100 purified Disassembly < 1 mg/mL | 22 |
| **RmmH** | Myc. smegmatis | TEM | **Nanotubes** | Not done |  | 4 |
| **RmmH** | Myc. smegmatis | TEM | **Nanotubes** | Not done |  | 1 |
| **RmmH** | Myc. smegmatis | TEM | **Nanotubes** | **Nanotubes** | cleavage of  SUMOylated construct | 9 |

(1) Young, E. J.; Sakkos, J. K.; Huang, J.; Wright, J. K.; Kachel, B.; Fuentes-Cabrera, M.; Kerfeld, C. A.; Ducat, D. C. Visualizing in Vivo Dynamics of Designer Nanoscaffolds. *Nano Lett* **2020**, *20* (1), 208-217. DOI: 10.1021/acs.nanolett.9b03651.

(2) Garcia-Alles, L. F.; Lesniewska, E.; Root, K.; Aubry, N.; Pocholle, N.; Mendoza, C. I.; Bourillot, E.; Barylyuk, K.; Pompon, D.; Zenobi, R.; et al. Spontaneous non-canonical assembly of CcmK hexameric components from β-carboxysome shells of cyanobacteria. *PLoS One* **2017**, *12* (9), e0185109. DOI: 10.1371/journal.pone.0185109.

(3) Dryden, K.; Crowley, C.; Tanaka, S.; Yeates, T.; Yeager, M. Two-dimensional crystals of carboxysome shell proteins recapitulate the hexagonal packing of three-dimensional crystals. *Protein Science* **2009**, *18* (12), 2629-2635, Article. DOI: 10.1002/pro.272.

(4) Young, E. J.; Burton, R.; Mahalik, J. P.; Sumpter, B. G.; Fuentes-Cabrera, M.; Kerfeld, C. A.; Ducat, D. C. Engineering the Bacterial Microcompartment Domain for Molecular Scaffolding Applications. *Front Microbiol* **2017**, *8*, 1441. DOI: 10.3389/fmicb.2017.01441.

(5) Kerfeld, C. A.; Sawaya, M. R.; Tanaka, S.; Nguyen, C. V.; Phillips, M.; Beeby, M.; Yeates, T. O. Protein structures forming the shell of primitive bacterial organelles. *Science* **2005**, *309* (5736), 936-938. DOI: 10.1126/science.1113397.

(6) Keeling, T. J.; Samborska, B.; Demers, R. W.; Kimber, M. S. Interactions and structural variability of β-carboxysomal shell protein CcmL. *Photosynth Res* **2014**, *121* (2-3), 125-133. DOI: 10.1007/s11120-014-9973-z.

(7) Sutter, M.; Faulkner, M.; Aussignargues, C.; Paasch, B. C.; Barrett, S.; Kerfeld, C. A.; Liu, L. N. Visualization of Bacterial Microcompartment Facet Assembly Using High-Speed Atomic Force Microscopy. *Nano Lett* **2016**, *16* (3), 1590-1595. DOI: 10.1021/acs.nanolett.5b04259.

(8) Lassila, J. K.; Bernstein, S. L.; Kinney, J. N.; Axen, S. D.; Kerfeld, C. A. Assembly of robust bacterial microcompartment shells using building blocks from an organelle of unknown function. *J Mol Biol* **2014**, *426* (11), 2217-2228. DOI: 10.1016/j.jmb.2014.02.025.

(9) Hagen, A. R.; Plegaria, J. S.; Sloan, N.; Ferlez, B.; Aussignargues, C.; Burton, R.; Kerfeld, C. A. In Vitro Assembly of Diverse Bacterial Microcompartment Shell Architectures. *Nano Lett* **2018**, *18* (11), 7030-7037. DOI: 10.1021/acs.nanolett.8b02991.

(10) Sutter, M.; Laughlin, T. G.; Sloan, N. B.; Serwas, D.; Davies, K. M.; Kerfeld, C. A. Structure of a Synthetic *β*-Carboxysome Shell. *Plant Physiol* **2019**, *181* (3), 1050-1058. DOI: 10.1104/pp.19.00885.

(11) Heldt, D.; Frank, S.; Seyedarabi, A.; Ladikis, D.; Parsons, J. B.; Warren, M. J.; Pickersgill, R. W. Structure of a trimeric bacterial microcompartment shell protein, EtuB, associated with ethanol utilization in Clostridium kluyveri. *Biochem J* **2009**, *423* (2), 199-207. DOI: 10.1042/BJ20090780.

(12) Schmidt-Dannert, S.; Zhang, G.; Johnston, T.; Quin, M. B.; Schmidt-Dannert, C. Building a toolbox of protein scaffolds for future immobilization of biocatalysts. *Appl Microbiol Biotechnol* **2018**, *102* (19), 8373-8388. DOI: 10.1007/s00253-018-9252-6.

(13) Pitts, A. C.; Tuck, L. R.; Faulds-Pain, A.; Lewis, R. J.; Marles-Wright, J. Structural insight into the Clostridium difficile ethanolamine utilisation microcompartment. *PLoS One* **2012**, *7* (10), e48360. DOI: 10.1371/journal.pone.0048360.

(14) Choudhary, S.; Quin, M. B.; Sanders, M. A.; Johnson, E. T.; Schmidt-Dannert, C. Engineered protein nano-compartments for targeted enzyme localization. *PLoS One* **2012**, *7* (3), e33342. DOI: 10.1371/journal.pone.0033342.

(15) Parsons, J. B.; Frank, S.; Bhella, D.; Liang, M.; Prentice, M. B.; Mulvihill, D. P.; Warren, M. J. Synthesis of empty bacterial microcompartments, directed organelle protein incorporation, and evidence of filament-associated organelle movement. *Mol Cell* **2010**, *38* (2), 305-315. DOI: 10.1016/j.molcel.2010.04.008.

(16) Havemann, G. D.; Sampson, E. M.; Bobik, T. A. PduA is a shell protein of polyhedral organelles involved in coenzyme B(12)-dependent degradation of 1,2-propanediol in Salmonella enterica serovar typhimurium LT2. *J Bacteriol* **2002**, *184* (5), 1253-1261. DOI: 10.1128/JB.184.5.1253-1261.2002.

(17) Uddin, I.; Frank, S.; Warren, M. J.; Pickersgill, R. W. A Generic Self-Assembly Process in Microcompartments and Synthetic Protein Nanotubes. *Small* **2018**, *14* (19), e1704020. DOI: 10.1002/smll.201704020.

(18) Kennedy, N. W.; Ikonomova, S. P.; Slininger Lee, M.; Raeder, H. W.; Tullman-Ercek, D. Self-assembling Shell Proteins PduA and PduJ have Essential and Redundant Roles in Bacterial Microcompartment Assembly. *J Mol Biol* **2021**, *433* (2), 166721. DOI: 10.1016/j.jmb.2020.11.020.

(19) Pang, A.; Frank, S.; Brown, I.; Warren, M. J.; Pickersgill, R. W. Structural insights into higher order assembly and function of the bacterial microcompartment protein PduA. *J Biol Chem* **2014**, *289* (32), 22377-22384. DOI: 10.1074/jbc.M114.569285.

(20) Lee, M. J.; Mantell, J.; Hodgson, L.; Alibhai, D.; Fletcher, J. M.; Brown, I. R.; Frank, S.; Xue, W. F.; Verkade, P.; Woolfson, D. N.; et al. Engineered synthetic scaffolds for organizing proteins within the bacterial cytoplasm. *Nat Chem Biol* **2018**, *14* (2), 142-147. DOI: 10.1038/nchembio.2535.

(21) Jorda, J.; Leibly, D. J.; Thompson, M. C.; Yeates, T. O. Structure of a novel 13 nm dodecahedral nanocage assembled from a redesigned bacterial microcompartment shell protein. *Chem Commun (Camb)* **2016**, *52* (28), 5041-5044. DOI: 10.1039/c6cc00851h.

(22) Noël, C. R.; Cai, F.; Kerfeld, C. A. Purification and Characterization of Protein Nanotubes Assembled from a Single Bacterial Microcompartment Shell Subunit. *Advanced Materials Interfaces* **2016**, *3* (1), 1500295. DOI: 10.1002/admi.201500295.
