# Supplementary material for: Inferring assembly-curving trends of bacterial micro-compartment shell hexamers from crystal structure arrangements
Source: PLoS Comput Biol. 2023 Apr 5;19(4):e1011038. doi: 10.1371/journal.pcbi.1011038 (PMC10109471; doi:10.1371/journal.pcbi.1011038)
Supplement: S4 Table — PDB codes are colored according to the type of organization in original structures that served to prepare the starting model for MD simulations (see S9 Fig for further details). For other details on how measurements were carried out, please refer to S3 Table. Please notice that local structural distortions might occur around residues selected for calculation of angles, which could result in misleading angle values. Such alterations might be amplified in the context of reconfigured interfaces. Angles therefore need to be contrasted with plane representations prepared taking the center of mass (COM) of hexamers (S9 Fig). (DOCX) [file pcbi.1011038.s004.docx]

**Table S4 – Structural changes during MDs trajectories when the starting tri-hexamer is reconfigured by positioning individual BMC-H hexamers as in *Arr-A* mode.**

| **Protein** | **PDBid** | **MD #** | **Tilting** | | | **Bending** | | | **Distance** | |
| --- | --- | --- | --- | --- | --- | --- | --- | --- | --- | --- |
|  |  |  | **Deg** | **(+/-)** | **Aver** | **Deg** | **(+/-)** | **Aver** | **Å** | **Aver** |
| **CcmK1*^6803^*** | **3BN4** | **1** | 1,0 | 4,8 | **0,0** | -21,9 | 9,2 | **-21,1** | -1,1 | **-1.1** |
|  |  | **2** | -0,9 | 3,6 |  | -20,3 | 6,3 |  | -1,1 |  |
| **CcmK2*^6803^*** | **2A1B** | **1** | 0,3 | 5,4 | **1,3** | -18,4 | 5,0 | **-21,2** | 0,5 | **0.3** |
|  |  | **2** | 2,3 | 3,8 |  | -24,1 | 6,6 |  | 0,2 |  |
| **CcmK2*^7942^*** | **4OX7** | **1** | 4,2 | 6,3 | **4,0** | -11,5 | 9,1 | **-11,9** | 1,9 | **1.8** |
|  |  | **2** | 3,8 | 6,1 |  | -12,4 | 7,0 |  | 1,7 |  |
| **CcmK4*^6803^*** | **6SCR** | **1** | 2,0 | 4,2 | **2,9** | -4,8 | 4,5 | **-2,4** | 1,8 | **1.9** |
|  |  | **2** | 3,8 | 4,5 |  | -0,0 | 3,6 |  | 1,9 |  |
| **CcmK4*^6803^***  ***pentamutant*** | **6SCR** | **1** | -0.1 | 2,6 | **0,5** | -14,0 | 4,4 | **-14,7** | 1,1 | **1.1** |
|  |  | **2** | 0.9 | 3,5 |  | -15,4 | 4,8 |  | 1,1 |  |
| **CcmK4*^7942^*** | **4OX6** | **1** | -3,6 | 4,8 | **-2,2** | -21,1 | 5,4 | **-20,9** | -0.0 | **-0.1** |
|  |  | **2** | -0,8 | 2,8 |  | -20,8 | 7,0 |  | -0.2 |  |
| **EutM*^Ecol^*** | **3MPW** | **1** | 2,4 | 4,8 | **1,6** | -27,6 | 7,0 | **-23,6** | -0.2 | **0.1** |
|  |  | **2** | 0,9 | 9,4 |  | -19,7 | 9,1 |  | 0.4 |  |
| **BMC-H*^Hoch^*** | **5DJB** | **1** | -0,7 | 2,9 | **-0.2** | -28,8 | 7,5 | **-29,5** | -1.2 | **-1.2** |
|  |  | **2** | 0,4 | 2,9 |  | -30,2 | 6,1 |  | -1.3 |  |
| **RMM-H*^Smeg^*** | **5L38** | **1** | -3,1 | 4,9 | **-3,1** | -28,3 | 8,1 | **-27,6** | -1.1 | **-1.1** |
|  |  | **2** | -3,2 | 3,0 |  | -26,8 | 6,6 |  | -1.0 |  |

PDB codes are colored according to the type of organization in original structures that served to prepare the starting model for MD simulations (see Fig. S9 for further details). For other details on how measurements were carried out, please refer to Table S3. Please notice that local structural distortions might occur around residues selected for calculation of angles, which could result in misleading angle values. Such alterations might be amplified in the context of reconfigured interfaces. Angles therefore need to be contrasted with plane representations prepared taking the center of mass (COM) of hexamers (Fig. S9).
