# Supplementary figures and images for: Inferring assembly-curving trends of bacterial micro-compartment shell hexamers from crystal structure arrangements
Source: PLoS Comput Biol. 2023 Apr 5;19(4):e1011038. doi: 10.1371/journal.pcbi.1011038 (PMC10109471; doi:10.1371/journal.pcbi.1011038)

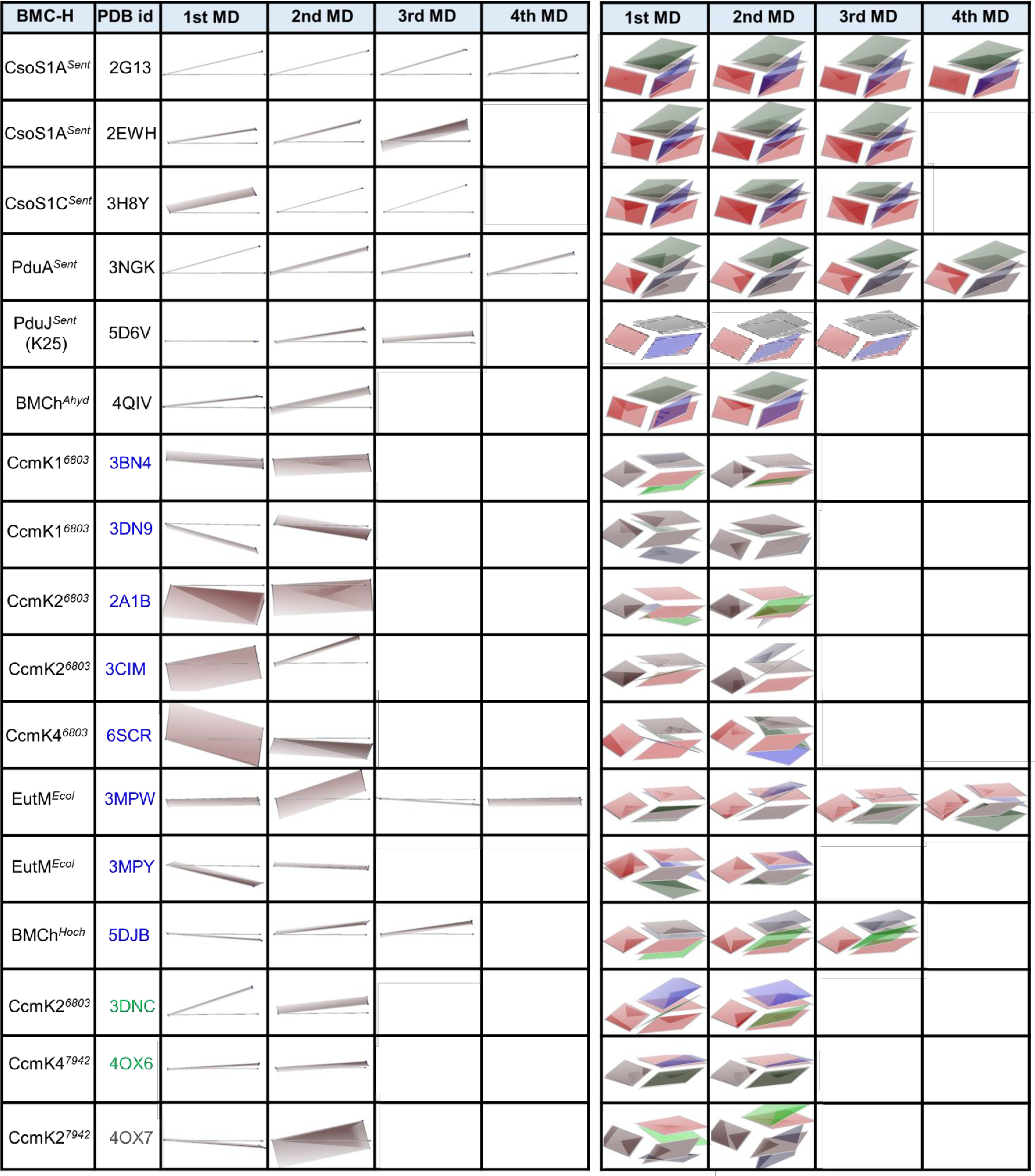

Supplement: S1 Fig — Two type of illustrations are presented on the left or right sides. They were prepared following the scheme explained in Fig 2B. PDB codes are colored according to the type of organization: black for Arr-A arrangements, blue for Arr-B, green for Arr-C and grey for Arr-D. (TIF) [file pcbi.1011038.s005.tif]

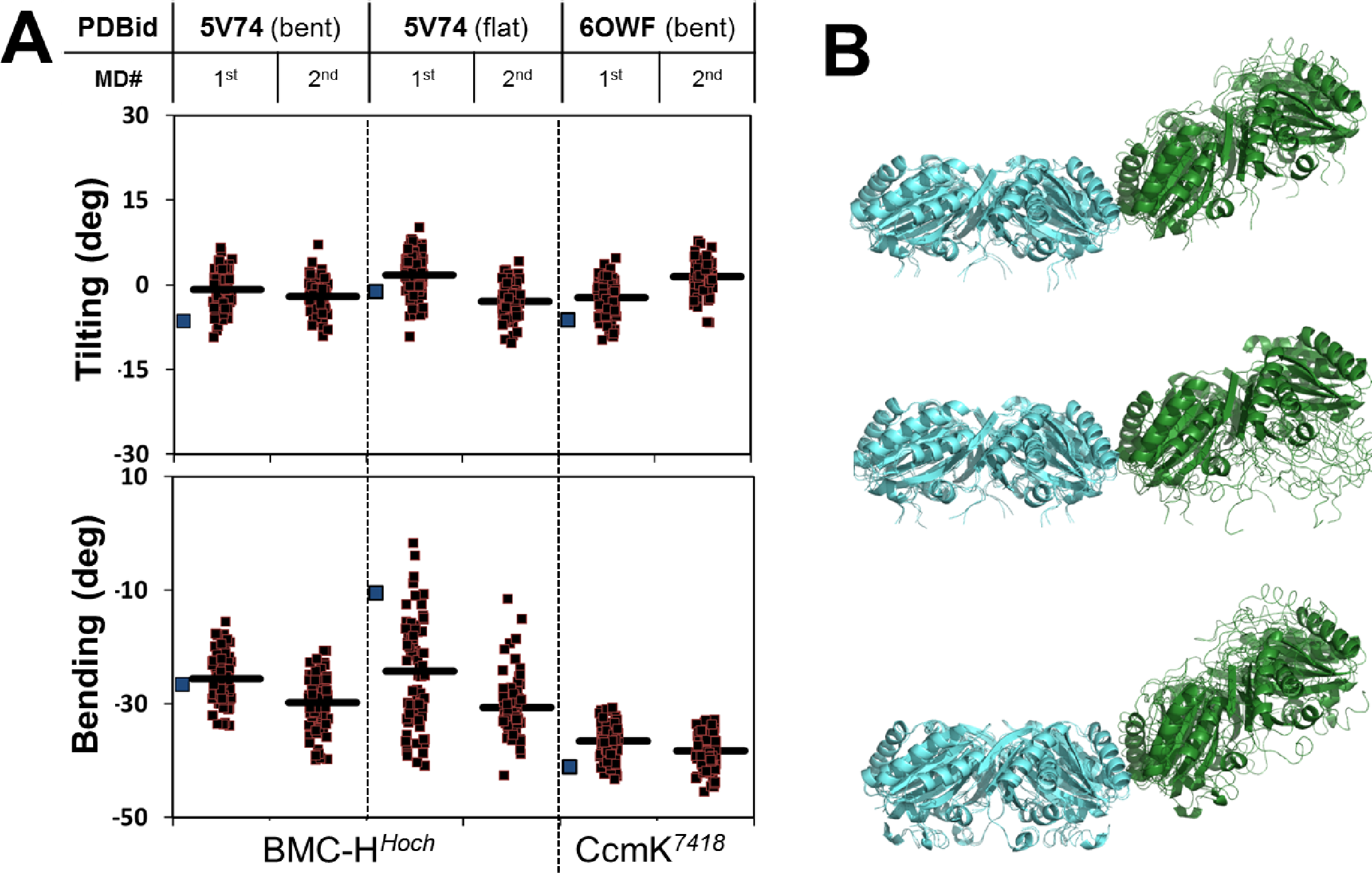

Supplement: S2 Fig — A, All-atom molecular dynamics of an ensemble of two hexamers extracted from published structures (PDBid codes indicated on top) and positioned in planar or curved configurations depending on the selected shell environment. See Fig 2 for further details. B, Comparison of structures generated by averaging atom positions over the MD snapshots (cartoons) with the starting structure (thin traces). The two structures were superposed on backbone atom coordinates of one of the hexamers (shown in cyan, on the left side). From top to bottom: bent BMC-HHoch (5V74), flat BMC-HHoch (5V74) and bent CcmK7418 (6OWF). (TIF) [file pcbi.1011038.s006.tif]

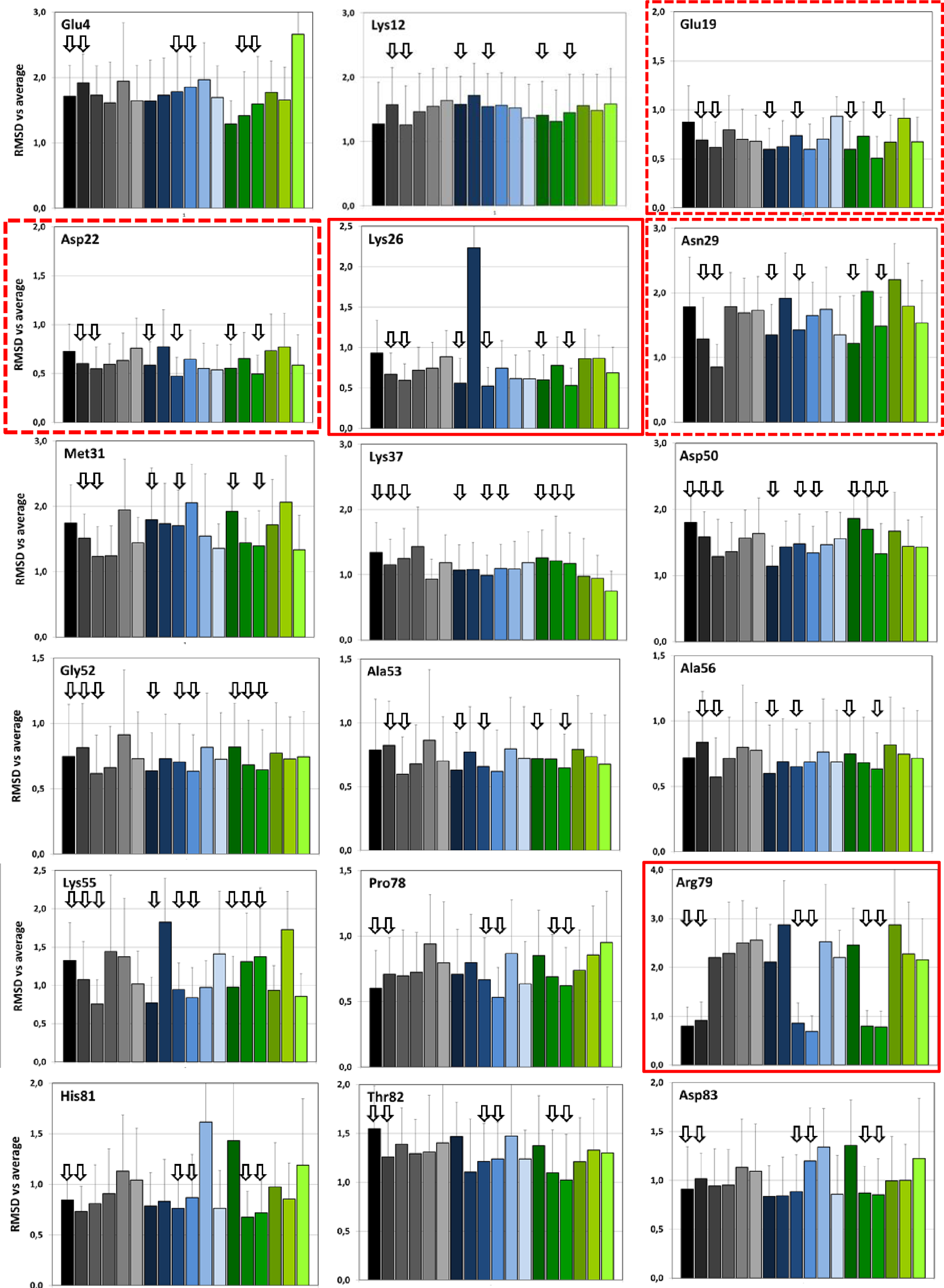

Supplement: S3 Fig — Represented is the average of root-mean-square deviations (RMSD) measured between side-chain atoms of indicated residues in each MD snapshot when compared to the residue atom coordinates in the structure averaged over the first PduA MD run. The first 8 snapshots were excluded from the calculations. Each panel present the values for a given residue in each of the 18 monomers of the tri-hexamer. Values in monomers from the first hexamer are shown in black to light grey scale, from the second hexamer with blue tonalities, green for the third. The arrows are to indicate residues from monomers that enter in contact with a neighboring hexamer. Similar results were obtained from data collected in the second MD run. Residues outlined in continuous red systematically show lower RMSD when placed at the inter-hexamer interface, in independent MD runs. Discontinuous outlines are for those residues that occur often, but not always, with lower RMSD. (TIF) [file pcbi.1011038.s007.tif]

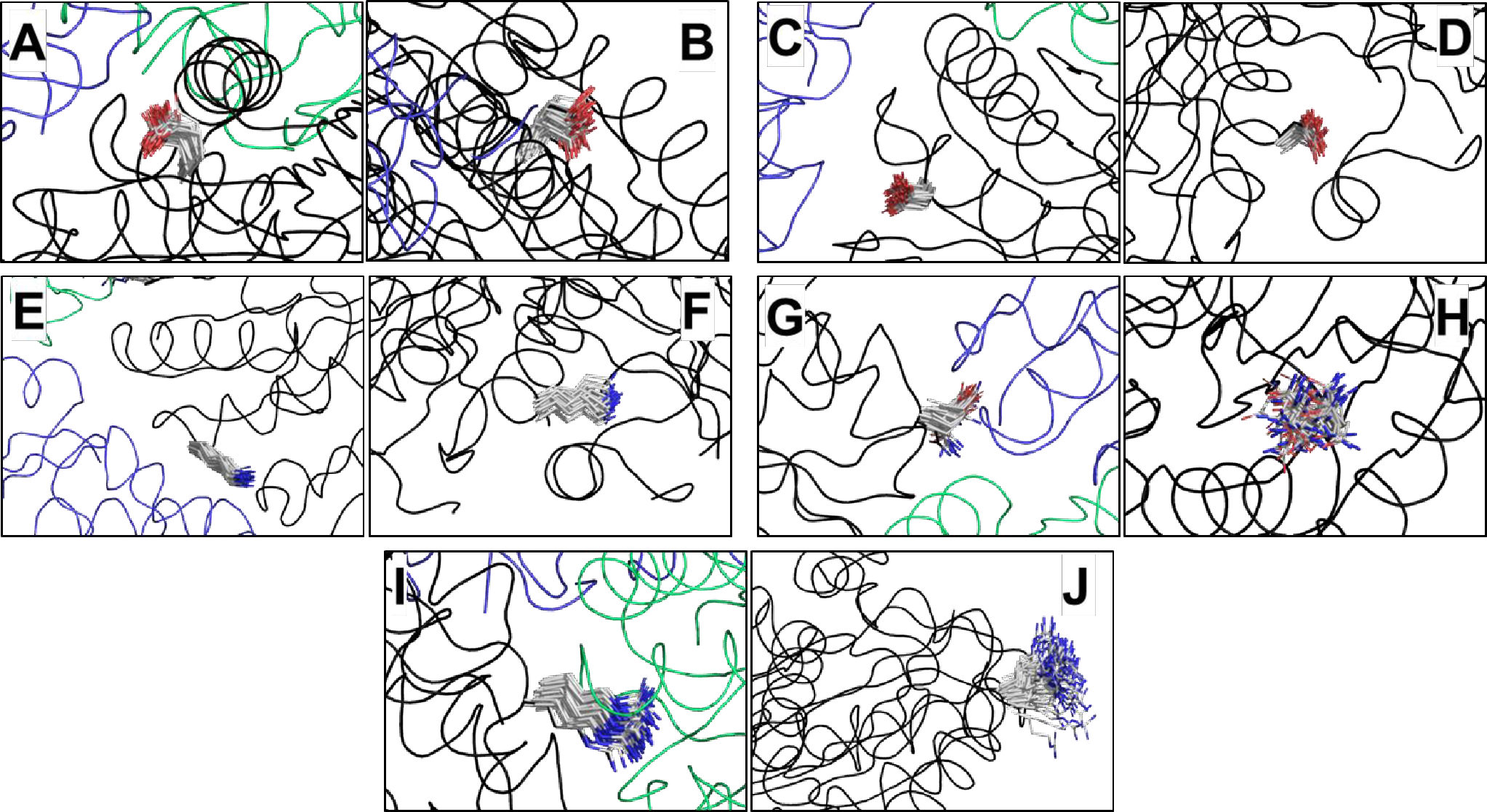

Supplement: S4 Fig — The view presents the side-chain conformations adopted by the several residues in the different collected snapshots of the first MD run on PduASent, depending on whether the residue lies at the inter-hexamer interface (left panels) or not (right): A-B: Glu19; C-D: Asp22; E-F: Lys26; G-H: Asn29; I-J: Arg79. Side-chains are represented as sticks, with nitrogens blue and oxygens in red. Residues were selected from data presented in S3 Fig. All snapshot structures were superimposed on main-chain atoms of one of the hexamers (black cartoon). The two other hexamers are shown in blue or green traces. (TIF) [file pcbi.1011038.s008.tif]

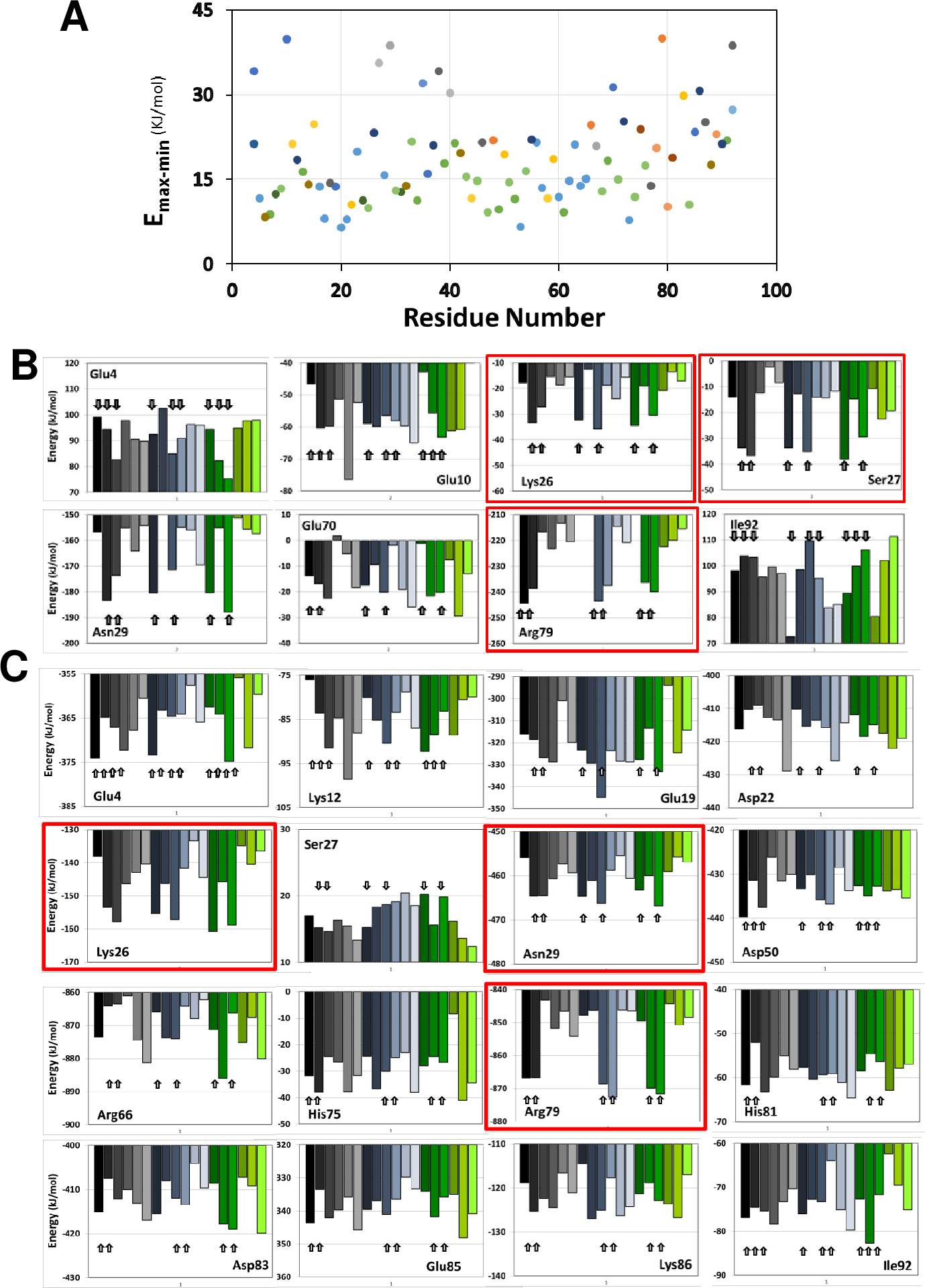

Supplement: S5 Fig — A, Interval of energies contributed by every residue of PduA, when comparing the 18 monomers of the tri-hexamer assembly. The ordinate presents the energy interval measured between the less and most stabilizing position. The most similar (lowest RMSD) snapshot to the averaged structure of the first MD run was selected for the analysis and energy computation was done with GROMOS96 implemented in Swiss-PDBViewer. B, Estimated energy contribution of selected residues in the 18 different emplacements of the trihexamer. Only a few residues among those analyzed are presented. Data are colored as in S3 Fig. The arrows are to identify residues in monomers that lie at the interface with neighbor hexamers. Outlined in red are residues that contribute a stabilizing effect when positioned at the inter-hexamer interface, for both MD runs. Similar results were obtained from data collected in the second MD run. C, Lys26, Asn29 and Arg79 were identified as stabilizing residues from plots of potential energy contributions averaged over the snapshots that covered the entire MD run. Here, potential energies were calculated using Amber (ff14SB) forcefield implemented in Yasara, and a single MD run was analyzed. (TIF) [file pcbi.1011038.s009.tif]

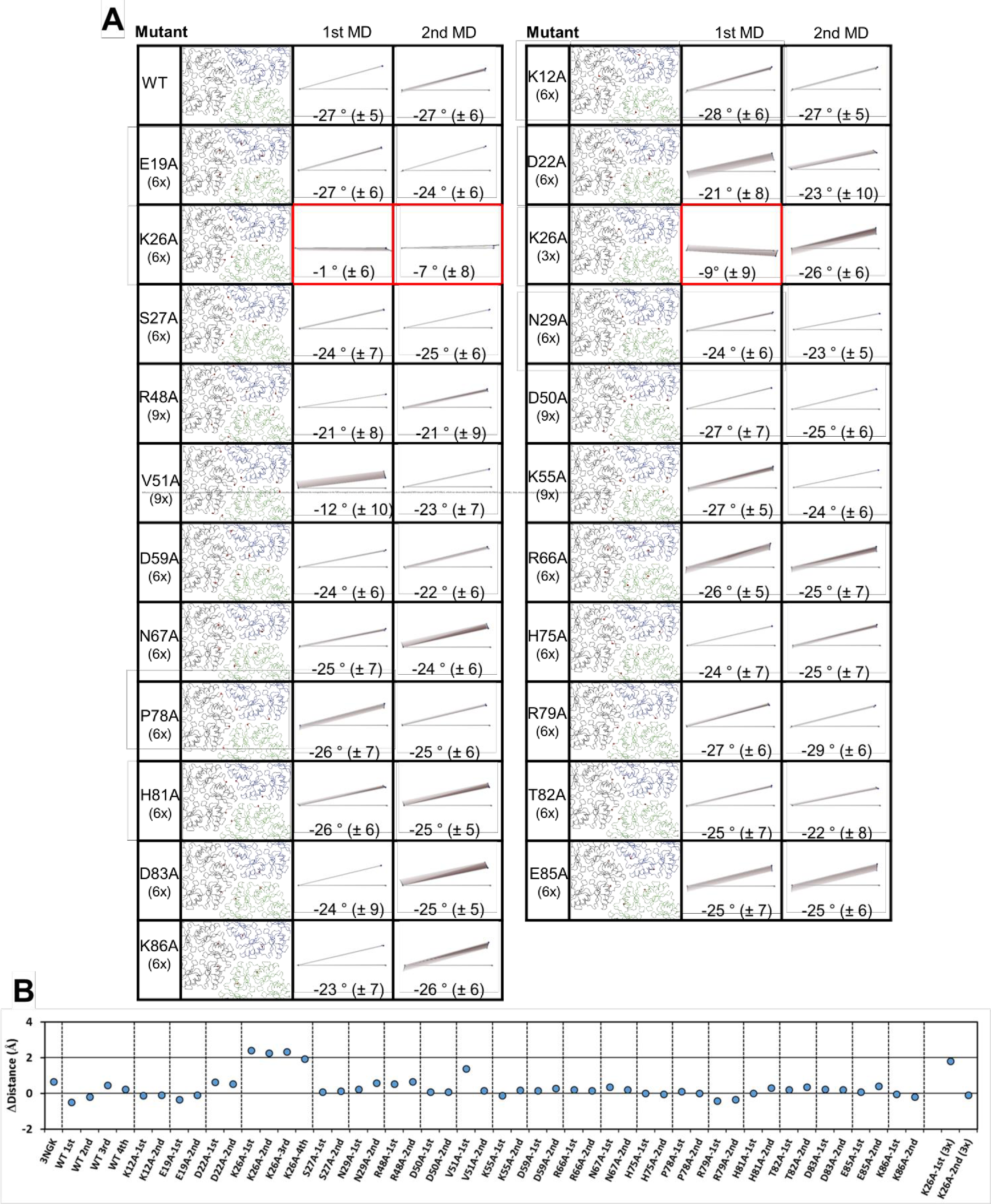

Supplement: S6 Fig — A, Residues indicated in the first lane were replaced by alanine in the 6 or 9 monomers of the trihexamer assembly that lie at the interface. An assembly with only three K26 positions mutated was also simulated. The Cα of such residues is indicated by red spheres in the second column. The result of two MD runs is presented following plane representations like those of Fig 3. Indicated bending angle values were evaluated like in S3 Table. Please notice that local distortions caused during the MD might in occasions result in under/overestimations. In the case of the K26A mutant (6x, outlined in red), four MD runs were carried out, with similar qualitative results. B, Effect of mutation on the distance between hexamers during the MD run. In the ordinate axes is represented the difference between the averaged distance calculated for the three hexamers (center of masses) in the averaged structure of a given MD simulation, and the average distance calculated from four independent MD runs on wild-type (WT) PduA, which are shown on the most left side, following the value measured for the PduA crytal (3NGK). Data obtained in independent MD run repetitions are denoted by 1st and 2nd label extensions below the X-axis. (TIF) [file pcbi.1011038.s010.tif]

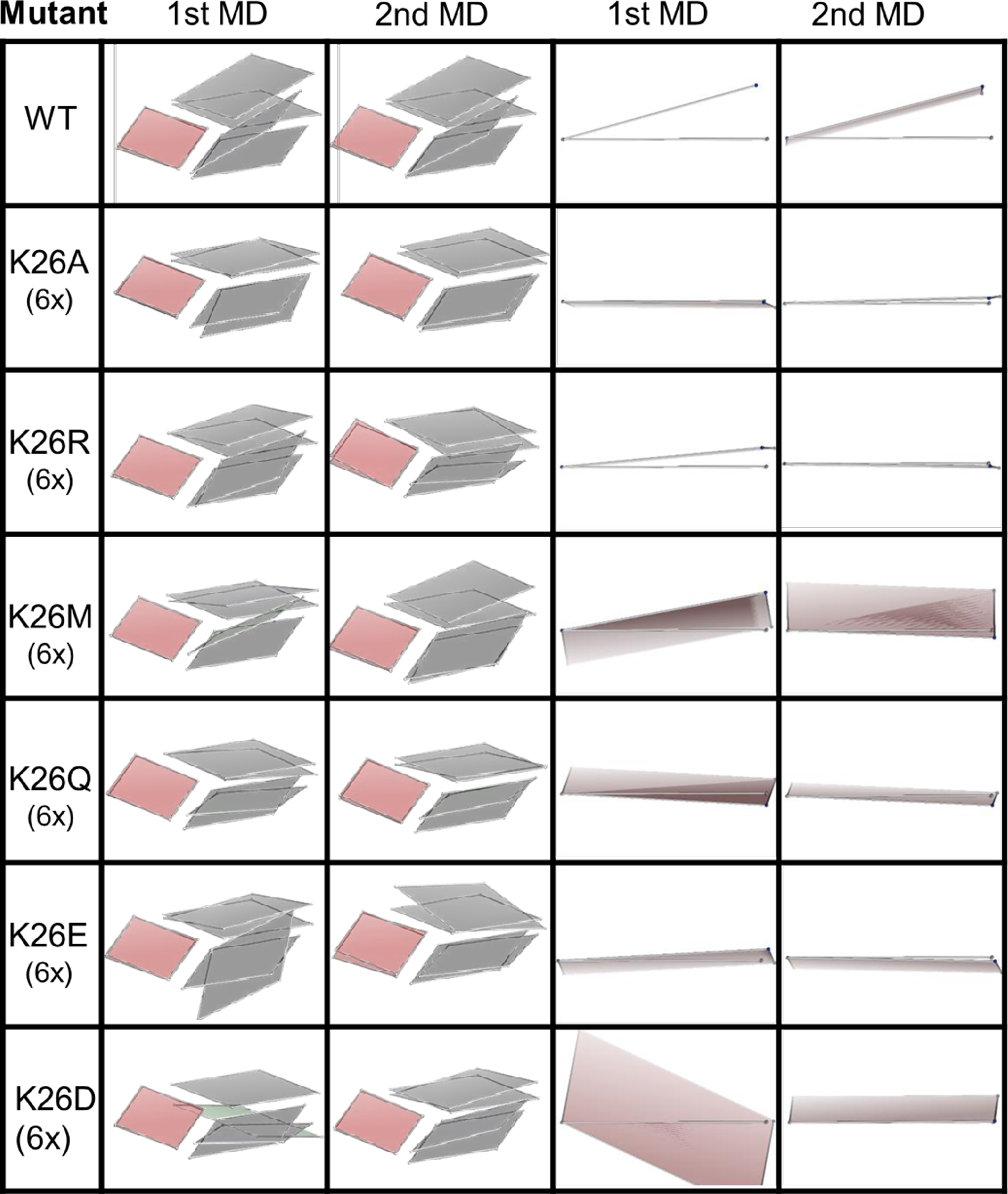

Supplement: S7 Fig — Lys26 was replaced by residues indicated in the first column in the 6 monomers located at the contacting interface between subunits. The result of two MD runs is presented following plane representations explained in Fig 2B (right side). (TIF) [file pcbi.1011038.s011.tif]

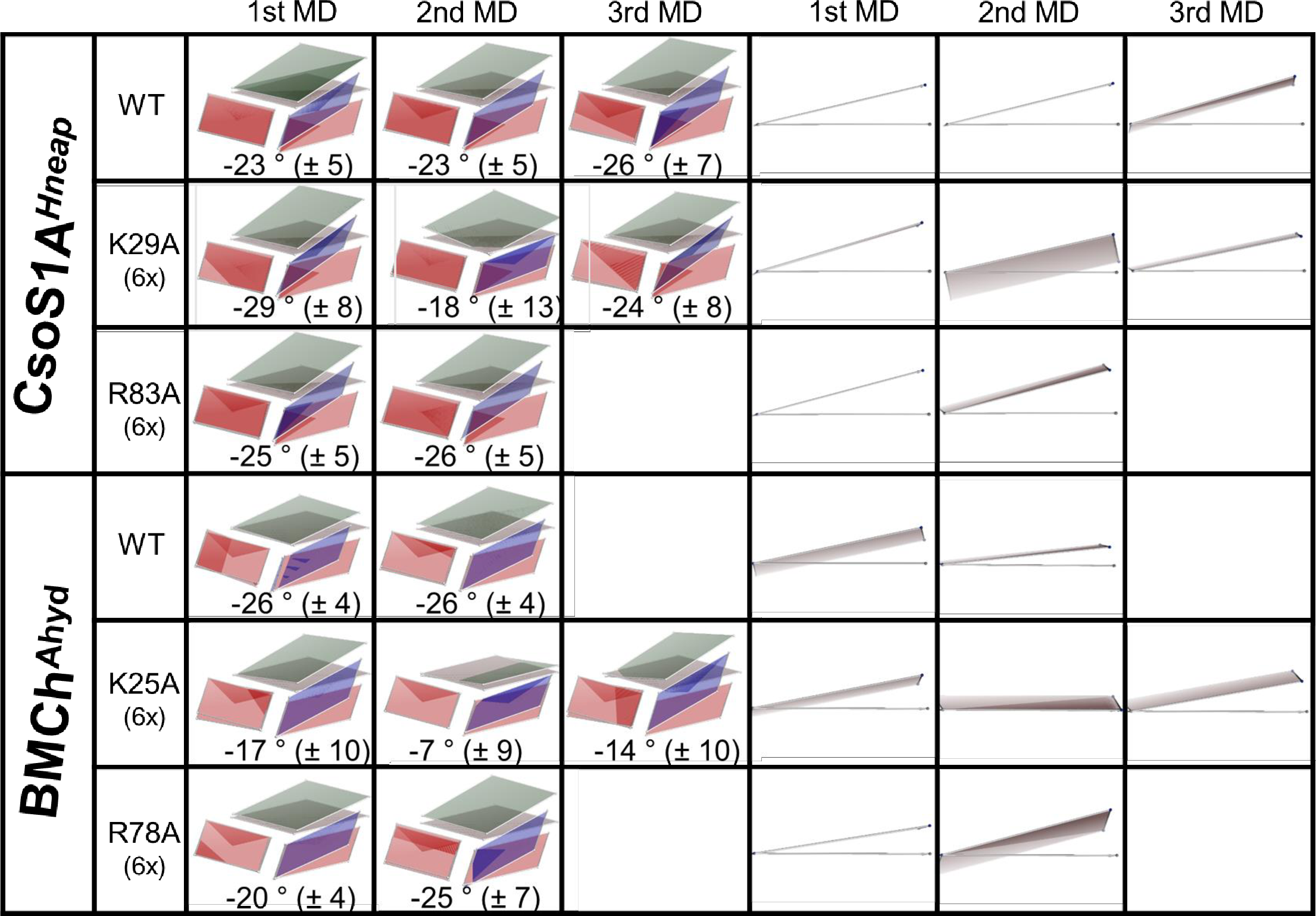

Supplement: S8 Fig — Key interfacial Lys and Arg, residues indicated in the second column, were replaced in CsoS1AHneap (2G13) or BMC-HAhyd (4QIV) by alanine in the 6 monomers of the tri-hexamer assembly that lie at the interface. Data from several independent MD runs are presented. Data for wild-type versions are shown for the ease of comparison. Hexamers are represented in the two ways presented in Fig 2B. Please notice that indicated average bending angles, calculated as explained in S3 Table, might be impacted by local displacements around the main-chain atom positions selected for the measurements. (TIF) [file pcbi.1011038.s012.tif]

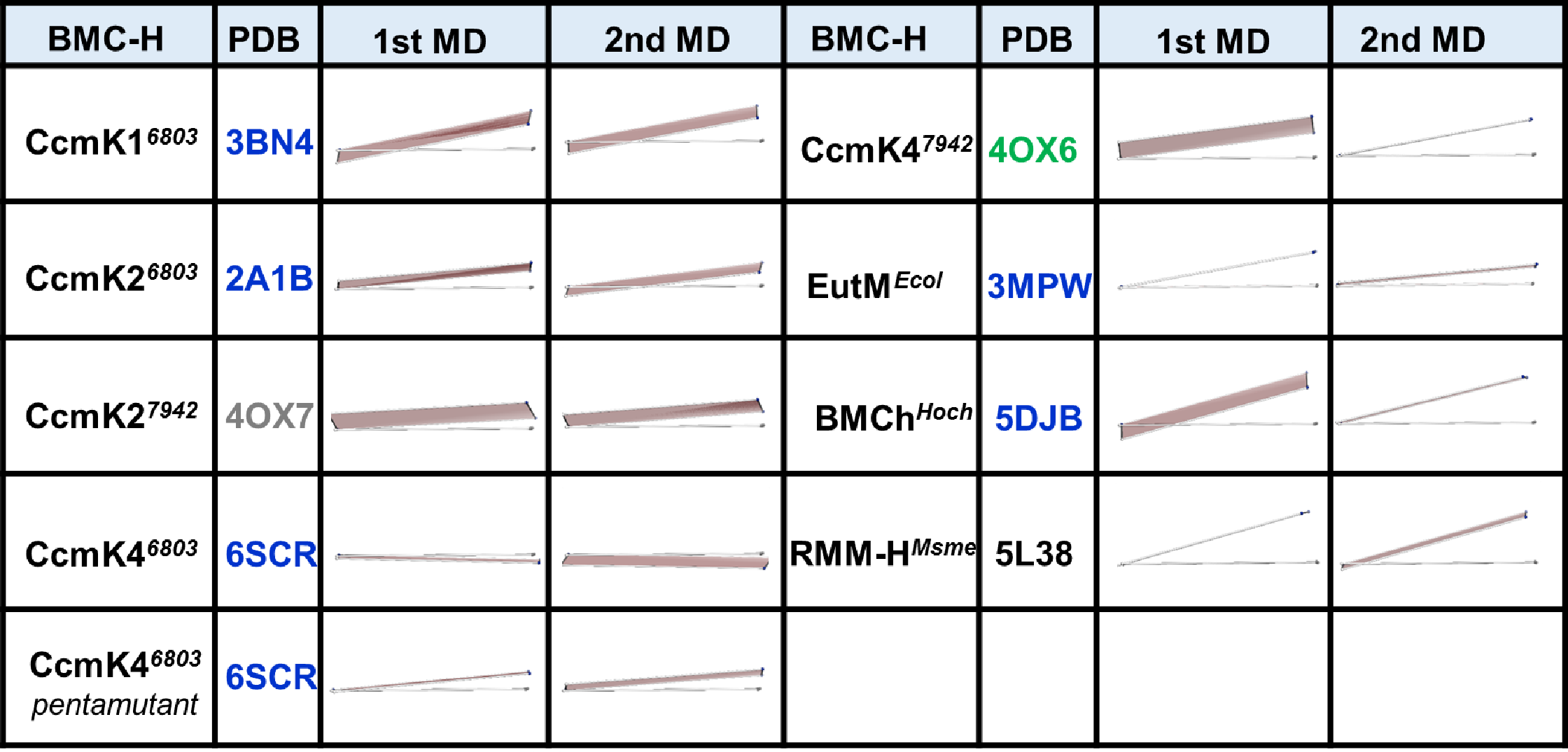

Supplement: S9 Fig — Comparison of the average structure generated for all snapshots of each MD simulation with the structure at time 0. The starting tri-hexamer ensemble was prepared by superimposition of an individual hexamer taken from the indicated PDB entry on each different hexamer of a template PduASent (3NGK) trihexamer, followed by manual interventions and energetic relaxation to prevent residue clashes (see M&M). Representations were prepared likewise in Fig 2B. PDB codes are colored according to the type of organization in the original PDB: blue for Arr-B, green for Arr-C and grey for Arr-D. Please note that RMM was included in the study, but the 5L38 entry do not belong to cases with tiled arrangements of hexamers. The CcmK46803 penta-mutant carried the next changes with regard to the WT version: R30N (6x), Q53G (9x), E54A (9x), E85T (6x) and N86D (6x). (TIF) [file pcbi.1011038.s013.tif]

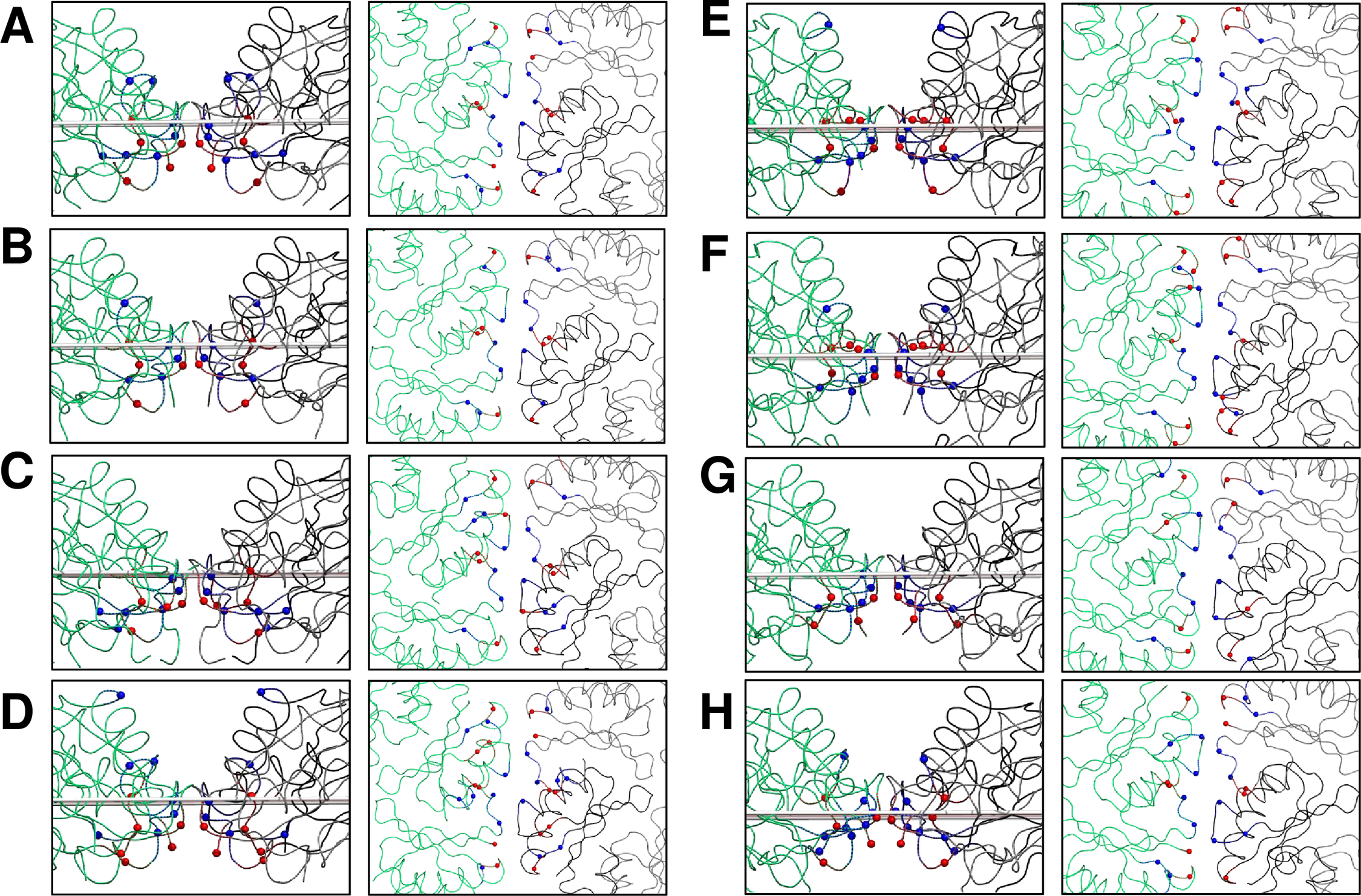

Supplement: S10 Fig — Represented is the localization of Cα atoms of cationic (Arg, Lys and His, blue spheres) or anionic residues (Asp, Glu, in red) around the interface of two hexamers. Only ionic residues lying closer than 7 Å from any atom of the hexamer counterpart are represented. Portions of each hexamer are depicted with green or grey traces, views being generated along the interface axis (left side of each panel, with concave side being at the bottom), or from top of the hexamers as seeing from the convex side (right). Left A-D panels correspond to Arr-A 3D structures: A, PduASent (3NGK); B, K25 PduJSent (5D6V); C, CsoS1AHneap (2G13); D, BMC-HAhyd (4QIV). Right panels are from Arr-B organized proteins, after reconfiguration of hexamers in Arr-A mode for the ease of comparison: E, CcmK16803 (3BN4); F, CcmK46803 (6SCR); G, BMC-HHoch (5DJB); H, EutMEcol (3MPW). (TIF) [file pcbi.1011038.s014.tif]
