# Supplementary material for: Inferring assembly-curving trends of bacterial micro-compartment shell hexamers from crystal structure arrangements
Source: PLoS Comput Biol. 2023 Apr 5;19(4):e1011038. doi: 10.1371/journal.pcbi.1011038 (PMC10109471; doi:10.1371/journal.pcbi.1011038)
Supplement: S1 File — (DOCX) [file pcbi.1011038.s015.docx]

The next three excel files are provided as SI:

1) ***MDsnapshot_angles_BMCh.xlsx*** contains raw measurements of tilting and bending angles measured for individual snapshot structures collected over MD runs on tri-hexamer assemblies **extracted from crystal structures**. These data are piled in columns D and E, and after being averaged in columns J and K over the 3 separated hexamer pairs (AB/AC/BC) of the different snapshots of each MD. These values can be read again in columns G, H of the *TABLE summary sheet* present in the same file, where they were compared to measurements established on the original crystal structures (columns C, D), thus permitting to evaluate in columns N and O the deviations indicated in Table S3.

Column F in the *DATA&Figures sheet* correspond to inter-hexamer distances calculated between identical atoms occupying same positions in each hexamer. These values therefore likely differ from distances indicated in Table S3, which were calculated from center of mass (COM) position of each hexamer in the MD average structure . These last values are indeed collected in the last columns of the TABLE summary sheet of the same file.

2) ***MDsnapshot_angles_BMCh_Arr-A_reconfigured.xlsx*** file similarly lists all values collected for snapshots from simulations on ***Arr-A* reconfigured** tri-hexamers . Data are presented following a similar organization as in the file presented above. The differences between angle average values for the MD and the starting reconfigured structure (time 0 of the MD) is presented however in columns O and P (same as in Table S4).

Distances calculated considering the COM of each hexamer of the MD average structure are given in the *Distances sheet*.

3) ***MDsnapshot_angles_BMCh_mutants.xlsx*** contains raw data from simulations on single point mutants, organized following the same scheme as for previous files. The number of mutations introduced per tri-hexamer are indicated.

In addition, several compressed file ***MD_input_files*** are furnished together with the YASARA macro script that was normally executed. Inputs are .pdb .pka and .sce files for each individual case, which were fed for the MD simulations. These files are organized in separated folders, to cluster together i) files related to tri-hexamer assemblies directly extracted from crystal structures, ii) rearranged in *Arr-A* configuration following the PduA 3NGK mode, or iii) those corresponding to mutants of PduA or of other BMC-H analyzed in this study.
