# Supplementary material for: Inferring assembly-curving trends of bacterial micro-compartment shell hexamers from crystal structure arrangements
Source: PLoS Comput Biol. 2023 Apr 5;19(4):e1011038. doi: 10.1371/journal.pcbi.1011038 (PMC10109471; doi:10.1371/journal.pcbi.1011038)
Supplement: Attachment — Submitted filename: Reply to Reviewers PCOMPBIOL-D-22-01712.docx [file pcbi.1011038.s016.docx]

Reviewer #1: Summary:

The authors of this manuscript study the spontaneous curvature of bacterial microcompartments (BMC) via analysis of constituent protein crystal structures, all-atom molecular dynamics simulation, and experiments. They survey a range of hexameric BMC proteins (BMC-H), demonstrating experimentally that most of the surveyed BMC-H assemble into structures of varying morphology which are much larger than the size of an individual hexamer. They then focus on 16 different BMC-H types for which crystal structures of their assemblies are available. The authors visualize and compare these crystal structures, paying special attention to the interface between hexamers, and identify different structural classes (“assembly types”) distinguished by the distances between key lysine residues at the inter-hexamer interface. Different types are associated with different experimental assembly morphologies (i.e. curved or flat.) To investigate this trend in more detail, the authors performed all-atom molecular dynamics simulations of trimers of hexamers of each kind of BMC-H. They found that hexamers of the Assembly A type reliably formed bent structures, consistent with spontaneous curvature in their large-scale assemblies, while the trimer structures of other BMC-H were much more variable. Further MD simulations of interacting hexamers in which key interfacial residues were mutated allowed the authors to identify key residues crucial for promoting spontaneous curvature. The authors also performed free energy calculations indicating that barriers separating different Assembly types in configuration space are small. However, simulations of trimers of non-Assembly type A hexamers initialized in an Assembly A configuration remained in this bent configuration for the duration of the simulations, suggesting that such configurations (which are consistent with a curved assembly morphology) may be metastable. Finally, the authors experimentally studied the effect of several mutations on the morphology of BMC-H assemblies, identifying key residues that promote Assembly A vs Assembly B structures.

Assessment:

The authors have performed a thorough investigation of the assembly morphologies of a large, representative sample of BMC-H proteins and the molecular features that lead to these different morphologies. While, as the authors point out, the precise microscopic mechanisms inducing spontaneous curvature of hexamer assemblies is still not entirely clear, the present work still significantly contributes to our understanding of BMC assembly and should be useful and interesting to scientists working on biological self-assembly. This is particularly important due to the fact that the origin (and even existence) of spontaneous curvature in microcompartment proteins is a significant (and contentious) issue within the field. Moreover, the potential switching mechanism elucidated here and its resemblance to confirmation all switching mechanisms and viral assembly points toward potentially generic features that biology employees for assembly of large icosahedral shells.

Main points:

●	In the “Atomic determinants for triggering BMC-H bending” subsection of “Results,” the authors describe a procedure for identifying contributions to the interaction energy between hexamers coming from different interfacial residues. I am a little confused why the authors choose only to look at the energies of one energy-minimized configuration. Is it not straightforward to extract the interaction energies for each frame in the MD simulation? The average over MD configurations seems like a more physically meaningful quantity to look at.

**We agree.**

**It is necessary to explain that this study was started several years ago. Although data presented in the MS are from about 100-150 independent MD runs, possibly more than double were in fact collected (studies of effect of pH, salts, residue insertions, etc). MDs were normally analyzed progressively, following the finalization of each run. Residue energy contributions were inspected occasionally in the described manner. Globally, analysis of such “average-like” snapshots coming from different dynamics recurrently showed the same conclusion: the major contributors are Lys26 and Arg79 (PduA numbering).**

**Despite time consuming, we verified reviewer’s concern. New data derived from MD-averaged values were collected and are qualitatively identical to those presented in Fig. S5 (corresponding to old Fig. S10). For instance, data for the two key contributors are presented here below:**

**Although absolute energy values plotted in the X-axes are different, likely as a consequence of using different force-fields for the analysis (GROMOS96 in Fig. S5, AMBER14 here), the same conclusion is reached: a consistent stabilization contributed by residues at inter-hexamer interfaces (indicated by arrows) was only observed for Lys26 and Arg79.**

**This is now mentioned in the legend of figure S5: “*Also important, the same conclusions were reached when plotted contributions corresponded to residue potential energies averaged over the entire 20 ns MD run, calculated using the AMBER14 forcefield in Yasara.”***

●	More details need to be provided for the umbrella sampling simulations. What was the bias potential used for each window? If it was a harmonic bias, what was the spring constant used, and on what value of the coordinate was it centered? How many configurations were sampled per window? Did the authors check that histograms between adjacent windows had reasonable overlap, and if so can they provide evidence of this? How was equilibration within windows and convergence assessed? I.e., if initialized from different initial conditions did they arrive at the same profile?

**Following the suggestion of both referees, we have added more details on the umbrella sampling simulations in the M&M section.**

**In reply to the specific questions raised by the referee, in each window a harmonic potential with a spring constant k=1000 kJ/mol/nm² was used as a biasing potential. The biasing potential was centered on different values of the order parameter equispaced at intervals of 1 Å. For each window, a NPT simulation of 2 ns was performed for equilibration, followed by a 10 ns NPT simulation sampled every 50 ps for producing the histogram that is then used to get the PMF. Equilibration in each window was corroborated by monitoring the convergence of the potential energy, typically reached well before the first nanosecond. Figure R.1 shows the potential energy in one of the 10ns production simulation (corresponding to the window centered at y=8 Å in the simulation for PduA*^Sent^* (3NGK)), evidencing that the system is fully equilibrated.**

**The values of the spring constant and the spacing between the center of the biasing potential were chosen to warrant a reasonable overlap between adjacent windows.  Fig. R2 shows the histograms used to reconstruct the PMF of PduA*^Sent^* (3NGK) shown in Fig. 4 (black line in the right panel). The simulations were repeated with two different initial conditions in each window, obtaining the same PMF profile within error bars, which proves a good convergence.**

**Fig. R1:** Value of the potential energy vs simulation time in one of the 10ns production simulation, corresponding to the window centered at y=8 Å in the simulation for PduA*^Sent^* (3NGK), showing that the configuration is fully equilibrated.

**Fig. R2:** Histograms obtained from the simulation and used to reconstruct the PMF of PduA*^Sent^* (3NGK) shown in Fig. 4 (right panel, black line). Each color represents the histogram obtained in a different umbrella sampling window with a harmonic bias potential with k= 1000 kJ/mold/nm^2^ and centered at intervals of y=1 Å.

●	Have the authors checked that the coordinates they refer to as “reaction coordinates” are actually reaction coordinates in that they can be used to identify transition states and have committor distributions peaked at ½? If not, please use the term “order parameter” instead (see Bolhuis et al., Annu. Rev. Phys. Chem. 2002 for a discussion of this point.)

**The COM coordinates “x” and “y” that we refer to as “reaction coordinates” are indeed not strictly “reaction coordinates” in the terminology suggested by Bolhuis et at, since we have not verified that they properly locate the right transition state with committor distribution peaked at ½. Please note that in this work we are not interested in locating the right transition state nor in measuring transition rates, but rather on reconstructing the Potential of Mean Force to identify the possible local minima representative of Arr-A, Arr-B, etc (using the new terminology). In this sense, the use of the term “order parameter”, as suggested by the referee, would seem more appropriate. But since we are not considering a phase transition and the coordinate that we are using to reconstruct the PMF is not any measure of “order” or “symmetry breaking”, the term “order parameter” is also not completely accurate. The terms “reaction coordinate” and “order parameter” are often used indistinguishably in umbrella sampling simulations, and in this case neither of them is fully precise. In any case, we have followed the suggestion of the referee and replaced the term “reaction coordinate” by “order parameter” in the manuscript.**

●	If Fig. 2C it is surprising how quickly the trajectories approach the bent state (within about one nano second according to the text). This seems like an unusually short timescale for such a large motion within a protein, which raises the concern that the initial structure was not sufficiently relaxed and incorporated. Can the authors give more details about their equilibration protocol and how they determined that relevant local degrees of freedom are relaxed within the initial state?

**The equilibration protocol was described in M&M. Basically, we first verified that no major problems were present in starting models, taken from the PDB entries. For most of cases, this was the case. When present, the problems were first resolved by trivial steepest descent minimizations in vacuo, before launching the Yasara protocol, which included a first phase of minimization in the presence of water/salts till convergence (<0.05 kJ/mol).**

**Please note that bending of the two hexamers does not require any large conformational change in any of the hexamers. It can be mostly considered a relative rotation between two almost rigid bodies driven by the lowering of the free energy in the cases where the bent state is the optimal arrangement.**

●	The authors might consider changing the “Ass-A” notation to something else, for two reasons: (1) Due to other uses in English, use of “Ass” is distracting; (2) the different classes (Ass-A, Ass-B, …) are defined based on distances between lysine residues at inter-hexamer interfaces, and not on the types of assemblies they form (although the authors show that these are correlated) – it may be more appropriate to call these something else, say “Class” A, B, …

**Yes, we did not realize about it. We have now replaced the terminology by this one: Arr-A, Arr-B, etc. It should now be better.**

●	In Figure 3, it would be useful to highlight the different protein names or PDBids based on their Assembly type, as the authors have done in Fig. S6.

**We agree. It is now presented following the same color scheme.**

●	Non-standard usage of some words can make the paper hard to follow. Here are some suggested changes of wording:

○	“Curved-implying” -> “curved” or “curvature-inducing” (l. 27, 107, 196)

○	“Conducting to” -> “leading to” (l. 31, 265, 425)

○	“Disposition” -> “position” or “conformation” or “pose” (l. 30, 33, 36, 56, …)

○	“Participate to” -> “participate in” (l. 22, 39-40, 60)

○	“Responsible of” -> “responsible for” (l. 34, 270)

○	“Permitted to” -> “permitted us to” (l. 28, 54, 129, 524)

○	“Challenged” -> “tested” (l. 227)

○	“Ensembles” -> “collections” or “pairs”/“triples” (of hexamers – is 3 really big enough to constitute an ensemble?) (l. 32, 109, 228, …)

**We introduced the mentioned changes.**

**We appreciated reviewer commentaries and acknowledge the effort in helping us to improve the clarity of the manuscript.**

Reviewer #2: Overview:

The paper proposed a novel self-assembly mechanism of BMC-H by inferring from AFM and TEM result and performing an all-atom MD simulation. The author provided fine detailed information of the interacting hexamers for a variety of BMC-H structures, which is further classified into 2 arrangements, Ass-A and Ass-B, characterizing by the distance between two key lysine residues. Classic MD simulations are performed in which the Ass-A type has seen strong and reproducible bending. However no common atomic feature has been found responsible for the BMC-H curvature, while a ionic pair between Arg/Lys are highlighted for maintaining the flatten Ass-B arrangement.

Some major questions:

1.	Different all-atom force fields were selected in classic MD (YASARA) and Umbrella Sampling MD (ff99SB-ILDN), why? A different selection of force field often results in different substates/subspace MD explored and will have non-negligible impact on PMF surface.

**The reason is that MD runs were carried out using different software (Yasara) than Umbrella sampling simulations (Gromacs). We agreed in selecting the AMBER force-field during discussions, but unfortunately, the most updated version of the AMBER force-field in GROMACS is not the same as in Yasara. However, ultimately, we believe that the fact that same bending trends (not shown in the MS) were observed with two slightly different versions should be considered as a guarantee of a more trustable results.**

**In line with that, it is important to mention that MD trajectories do not seem to change significantly when changing different versions of AMBER available within YASARA: AMBER99, AMBER11 and AMBER14. More surprising, the same result was obtained when the system was described with a CHARMM forcefield (carried out by the third team), something mentioned in the text.**

2.	In the section of umbrella sampling simulations, how the parameters were defined to conduct the steered MD? For example, how were the extent of biasing potential and number of umbrella windows selected/optimized? Will a different set of such parameters PMF surface?

**As indicated above in the reply to reviewer 1, we have now better detailed the protocol for umbrella sampling simulations in the M&M section.**

**In reply to the last specific question raised by Referee #2, the use of different sets of spring constants, total number of windows, or centering in the biasing potential do not affect the reconstructed PMF surface, provided that the different umbrella windows overlap.**

3.	What is the time scale of BMC self-assembly? Is 20ns run typical for observing such event?

**Please notice that our MDs did not simulate the process of assembly, which as the reviewer supposes, it is expected to require longer runs – the time scale of self-assembly is expected to depend exponentially on the concentration of hexamers and will typically require longer than 20ns for successful assembly – We instead just monitored relatively little displacements around starting pre-assembled structures taken from PDB entries.**

**Possibly informing on the time scale of the assembly process, hexamer dissociation insinuated in occasions. For instance, when starting structures were incorrectly relaxed. More interesting, in experiments with the PduA K26A mutant, the inter-hexamer distance always increased by 1.5 to 2 Å in the course of 20 ns MD (shown in Figure S6B).**

Some details to be changed:

1.	In MD analysis section, the term “main-chain atoms” is ambiguous and appeared several times without definition, clarify if this is backbone atoms, backbone C-alpha atoms or heavy atoms.

**We do not completely agree with the reviewer in considering main-chain atoms more ambiguous than backbone atoms. Nevertheless, we replaced the term for the present MS.**

2.	In Figure 1 caption: “… (2A1B) in pale green, CcmK46803 in limon, …)”, is ‘limon’ a typo?

**In fact, it is one of the color selections in Pymol, which is possibly the most common software for generation of protein structural pictures. That is why we preferred to keep the same terminology.**

3.	In line 443: “This is supported by…imaged by TEM.”: Please add the Fig.1 for referencing.

The author specified this Arg28/Asp49 is responsible for “Ass-B arrangement”, while in line 345 this is referred as “non-Ass-A organizations.” Please unify the terms here.

**These suggested changes are not relevant for the present version. The corresponding sections were removed, following the proposition of the Editor, which was based on commentaries from Reviewer 3.**

4.	In section “Multiple energy minima in lateral contacts between planar BMC-H”, few kBT is too ambiguous. Author should specify what are the energy barriers (and the initial/final state of the transition) explicitly in the same unit of energy barrier plotted as in kcal/mol.

**This unnecessary expression was removed from the text.**

a copy also uploaded as a word file.

**We thank the reviewer for helping us to improve the MS.**

Reviewer #3: In this manuscript, the authors studied the assembly and interaction properties of hexameric shell proteins of bacterial microcompartments from different bacterial origins, using protein expression, electron microscopy, atomic force microscopy, and all-atom molecular dynamics simulations. Based on the results, the authors proposed the possible mechanisms of shell hexamer self-assembly to form either curved tubular structures or flat sheets when expressing the shell hexamers in E. coli. Although the study offers some information about the assembly nature of bacterial microcompartment shells in general, some of the results have been reported in several previous publications, reducing the novelty of the study. Moreover, some results were not strongly supported by the microscopic or biochemical results, and the overall study was largely descriptive instead of conclusive in respect of the detailed mechanism. My specific concerns are listed below.

- Large structural variabilities of shell assemblies can often be observed in E. coli (in this work and previous studies), strongly depending on the expression conditions and protein expression levels. Therefore, the target protein abundance in different expression hosts and isolated protein samples should be precisely compared for analysis.

**Covering different expression hosts, possibly promoters, RBS, etc was not under the scope of this study. In that sense, we monitored in Fig. S1B total protein expression levels, solubility and recovered amounts. These data indicated that the level of expression were relatively comparable for almost all BMC-H, exception made of C-ter tagged CsoS1A, and to a lower extent of EutM and CcmK2^7942^.**

**On the contrary, isolated protein samples were quantified and, as mentioned in the text, assembly experiments were carried out at similar protein concentrations.**

- AFM and EM results were largely descriptive than conclusive. Some AFM and EM results of the same samples were also contradictory to each other. The descriptions and presentation of the microscopic results are also difficult to read.

**This point is treated below, since the reviewer did not mention here the source of such contradictory data.**

**With regard to the descriptions and presentation, the reviewer is possibly right. For the future manuscript presenting these and new experimental results, we will try to be more factual and provide better organized data. We apologize about it.**

- BMC-H proteins have high sequence and structural similarities. This manuscript seems to claim the possible mechanism of shell protein self-assembly behaviors based on the reported crystal data of BMC-H, though there are always exceptions in individual groups. In reality, one would not expect to have to achieve crystal data of proteins for predicting or understanding their assembly behaviors. It would be more informative if this work shows a case study by predicting the assembly of an uncharacterized protein using MD simulations and then applies microscopes to verify its assembly.

**It was not our intention to propose that it is necessary to get a crystal structure to predict assembly. Our intention is just to seek for potential connections between the way such crystals organize and the spontaneous curvature behavior of a given BMC-H.**

**We agree with regard to his/her advice of applying MD to predict assembly. This will constitute a portion of our future work.**

- The crystallographic conditions of PduA 3NGK is 2D tilling, but in E. coli expression hosts they form nanotubes with curved interactions. How much can the structural features gained from crystal data reflect the actual protein interactions in the nanotube-forming conditions?

**The 8 structures of recomposed BMC-shells indicate that the interactions drawn in crystals are basically maintained in the shell context. Although Pdu (or PduA) does not figure among characterized shells, it would be extremely surprising that assembly is not governed by the same set of interactions conserved for other BMC-H types.**

- The manner of tagging may have notably effects on the assembly as shown in previous studies. Have the authors tried the His-tag tagging on the N-ter or C-ter of BMC-H for comparison?

**We tried here only for two cases, CsoS1A*^Hneap^* and CcmK2^7942^. The reason is that a previous study of our group on CcmK proteins from Syn6803 (reference 23) indicated much lower yields of expression with N-ter tagged constructs.**

**We could not compare results on the protein CsoS1A, because expression levels were too disparate. However, flat structures formed with the N- and C-ter tagged CcmK2^7942^ constructs, as shown in Fig. S3D vs S3E.**

- The conformational changes or bending of individual hexamer structures compared to their crystal structures have often been identified during protein assembly in structural analysis, which is relevant to the bending of two neighboring hexamers. There is no description if the conformational changes of BMC-H were characterized in MD simulations.

**Unfortunately, we were unable to understand reviewer’s question/demand, which is somehow ambiguous.**

**Is he/she asking to measure RMSD deviations within hexamers, to give an indication of the extent of deformation of monomers or of hexamers that might be happening during MD? We did not monitor such values because, but globally speaking, changes are minor.**

Line 47-48: What is the evidence that support the random assembly of cargo and shells of Pdu compartments?

**Basically, an uncoupled emergence of signals deriving from assembly of shell proteins and cargo proteins simultaneously tagged with different fluorescent reporters.**

**This is mentioned in the introduction, including a citation of the original article: reference 13.**

Line 89-90: please specify what the scaffolding factors are.

**We now explicitly mentioned proteins considered to play scaffolding roles in Beta-carboxysomes: “*shells appearing only after the emergence of pre-organized procarboxysome “grains” of coalesced RuBisCO, carbonic anhydrase and scaffolding proteins like CcmM and CcmN*”**

Line 96-98: Has curvature been observed in native BMC shells that are composed of predominantly hexamers or only in hexamer-hexamer assemblies?

**We are not sure to understand the question.**

**Spontaneous curvature defines an intrinsic protein property that measures its propensity to assemble giving rise to curved objects.**

**Experimental data indicate that many BMC-H are prone to curve directly. However, all reconstituted shells so far characterized present BMC-H in bent configurations. Flat arrangements are also present in only a single case.**

Line 100: Pang et al. JBC 2014, 289(32): p22377-22384 and Uddin et al. Small 2018, 14 (19): 1704020 should be cited.

**We don't understand why these two studies, both from Pickersgill RW and collaborators, should be mentioned as antecedent of investigations of spontaneous curvature. They were in fact referenced and discussed in other sections of our manuscript (ref 17 of the main text, also ref 17 and 19 of Table S1). But they do not constitute investigations of spontaneous curvature.**

**Uddin *et al* dealt basically with how hexamers are oriented in PduA and PduB nanotubes. Models were prepared in the beginning of the article with the intention to predict the orientation sense. A concave-out organization was selected, based on the lower number of atomic clashes induced upon displacing with Python scripts the hexamers in that direction, as compared to the opposite one. However, atoms were not allowed to “adapt” and not even a relaxation of intermediate models was attempted. Should we consider this approach as precedent of studying curvature?**

**With regard to Pang *et al*. work, although published data valuably proved that mutation of K26 and R79 residues disrupted nanotube formation with PduA, we firmly disagree in considering it as a work dedicated to the investigation of spontaneous curvature.**

Line 126:.PduB was missing (Small 2018)

**The reviewer insists in citing Pickersgill’s work at an irrelevant place of our text.**

**Here, we listed BMC-H hits that were studied experimentally in our study. We did not study PduB. Besides, PduB is an exemple of BMC-T, and not BMC-H, which are the focus of this manuscript.**

Line 140: What are the sufficiently low concentrations? Please specify it here and cite references. How to determine if the presence of urea (even at low concentrations) will affect the assembly? How to differentiate BMC-H larger ordered assemblies and inclusion bodies?

**The information is not any longer necessary, since we removed the experimental data.**

**However, it is necessary to say that the information appeared in the sentence that came right afterwars: “*resolubilization of IB required at least 2 M urea concentrations”*. The precise amount was indicated in the M&M section (line 520), also in other section of the main text (line 354).**

Fig. S1: It would be useful to show the phase contrast microscopy images together with panel A. panel B, It is unclear how to define the assembly structures using this crude isolation protocol. Western blot is necessary to specifically identify the target proteins, since these proteins have been tagged with His-tags.

**It would have been possible to add the phase contrast images, although we did not see a clear interest.**

**In panel B we did not pretend to define assembly structures, but simply provide evidences suggesting that assembly in cells likely happens for most BMC-H. We considered as proofs of assembly material that are recovered soluble from pellets with low urea amounts (1M), concentrations that are insufficient to solubilize inclusion bodies.**

**We completely disagree in the need to identify target proteins by Western Blot. First, because BMC-H proteins are so strongly expressed in cells that they constitute the most abundantly detected protein in cells (see the cellular fractions, CF). Second, because the proteins are also purified, as shown in the corresponding portions of the figure (PF and UPF fractions). Why an antiHis6 antibody should work better in identifying a histag-protein than an IMAC column ?**

Line 162-163: The authors claimed earlier in the manuscript that no noticeable observations for CcmK2 proteins were observed in E. coli. Were the 2D flat organizations observed by AFM formed by in vitro assembly of isolated proteins?

**We wrote one sentence before, in line 161: *Assembly potential was also assessed by AFM and TEM taking purified proteins conditioned at comparable concentrations*. Could that be incorrectly written? Is it ambiguous?**

Fig. S3 showed low-resolution AFM images of CcmK “assemblies”. The limited resolution do not seem to support strongly the conclusion regarding the highly diverse forms of CcmK aggregates. Some images appeared to be captured with overloaded samples or contaminated probes even. It would be necessary to show higher resolution images which will allow one to identify individual hexamers and regular patterns in the 2D sheets.

**It was not our intention to convey an idea of highly diverse forms of CcmK aggregates. We are uncapable of finding a “sentence” that could led the reviewer to reach such conclusion.**

**In fact, our intention was to present images that CLEARLY prove that all CcmK assemble flat. In AFM, flat assemblies manifest as horizontal platforms or islands (color scales spreading a few nanometers), depending on protein load. We agree that the resolution is not high, and possibly not as well appreciated in the submitted figure as in the original ones. But we believe it should suffice to convince on the formation of flat assemblies. We dispose of dozens of images that proof that CcmK proteins are recurrently organizing flat above mica supports.**

Some AFM and EM observations of the same samples were also contradictory to each other. For example, CsoS1AHneap assemblies are small 2D fragments in AFM but form filaments.

**AFM is not an adequate technique to monitor rounded structures. This was the reason why we wrote in lines 154-156:”** ***Less clear-cut and difficult to reproduce data were obtained for the rest of BMC-H, pointing to difficulties expected if rounded objects formed, since these would establish weak contacts with the planar mica support*”.**

**In fact, a recurrent impossibility to monitor assemblies might indirectly point to curving phenomena (or aggregation, etc). For these cases, images vary from preparation to preparation, and even when moving to different sectors of the same sample. That is why we opted by presenting several images for each irreproducible case, contrasting with CcmK proteins, which reliably give rise to flat “islands and carpets”. We now understand we mistook in showing things that way.**

**That said, we do not consider as contradictory to observe two or more type of structures for the same protein.**

**What mattered to us was that, for instance, no flat structures were revealed by AFM with CsoS1A (mostly often spherical objects were seen), whereas TEM and cryo-EM revealed nano-wires, nanotubes and spheres, altogether attributing curving features to this BMC-H. Similarly for other BMC-H.**

Fig. S3C-E, S3J: How did the authors know these filament structures were BMC-H assemblies not contaminations during sample isolation or preparation for imaging?

**We assume the reviewer refers to Fig. S4 panels, and not S3.**

**Conclusions were extracted from the observation of similar structures in different preparations of the same protein, as well as because such structures were not seen in controls without proteins. An exception is the case of CsoS1A, for which we mentioned in the text (line 188) that “… *were* *detected in only one out of three independent preparations*”, to signify the possibility that such nanowires could not be representative.**

**We agree with the reviewer in being cautious, since these structures were seen rarely, and the verification of the composition was not carried out (for instance with antibodies).**

Why were some AFM images shown as dissipation mode views than height views?

**Because they provided a better resolved alternative view of the same objects. The objects appears similar in the normal view mode.**

Line 183: It would be difficult to exclude the possibility that the observed nanotubes on 2D tile edges was due to sample overlapping instead of 2D growth.

**It is possible that we did not understand what the reviewer means by overlapping.**

**Is the reviewer thinking of the possibility of capturing together in the image independent objects, located for instance at different sample depths? That seems statistically unlikely, as not only the image shows different nanotubes apposed on several edges, but also other images showing the same were collected.**

Line 199: add the references.

**The reviewer is right. We should have pointed to the Table S1.**

Line 219: what are CsoS1ASent and CsoS1CSent?

**It was a typing error. The 2EWH/2G13 and 3H8Ystructures correspond to CsoS1A and CsoS1C from *H. neapolitanus*.**

**We thank the reviewer for identifying the error. We are surprised by the way the question is written: could it be possible that the reviewer did not realize about the error? The correct attribution is mentioned 12 times in the text (lines 126, 144, 170, 188, 197, 212, 248, 258, 306, 335, 405, 415). The PDB codes are also indicated in the sentence, which should permit to clarify the doubt.**

Fig. 2: In the PduA nanotube, three neighbouring BMC-H bend only at the interface along the long-axis of the nanotube, which predominantly drives nanotube formation, instead of tilting towards the center of the three BMC-H as shown in Fig. 2B.

**Although geometrically speaking that seems reasonable, as far as we know, there is no experimental demonstration permitting to state so categorically the mechanism of formation. Theoretically, we are only aware of several studies using coase-grained models that successfully reproduced the formation of nanotubes from spherical representations of BMC-H subunits. Depending on the precise selection of parameters, nanotubes or spheroids were obtained, suggesting that transitions between the two structures should be feasible.**

Line 295-296: explain the scenario of making alanine mutants on the identified residues (in a chapter below) used some other residues in addition to Ala for genetic replacement)?

**We apologize, we do not completely understand…**

**We would be surprised that the reviewer asked to use residues other than Ala to investigate given positions. This would be an unusual risky approach, for several reasons that would be long to detail.**

**If he/she is referring to the study of K26, we would be even more surprised, since a paragraph of the MS presented the results of the replacement by other residue types (see Fig. S12, for instance, now Fig. S7)**

Line 367-369: It is not accurate to compare EM and AFM results directly for compactness analysis. The same technique should be exploited for the comparison.

**The reviewer is right. In future studies, we will try to compare both mutant and WT imaged by AFM.**

Line 418-419: How confident can we rule out the effects of different crystallization conditions on the protein packing in crystals? For this, one should expect to achieve experimentally the same crystal packing data of the same protein at different crystallization conditions.

**This is precisely what we mentioned in lines 217-221: “*the organization mode is reproduced in crystals of the same protein prepared under variable conditions (e.g. Ass-A for structures 2EWH and 2G13 from CsoS1A^Sent^, or in the 3H8Y structure of the close CsoS1C^Sent^ homolog; Ass-B for 3BN4 or 3DN9 structures of CcmK1^6803^ or 3MPW and 3MPY from EutM^Ecol^)”.***

Line 429-431: Please provide the comparison to reveal how similar they are.

**This was mentioned in Results section, lines 253-256: “*Indeed, root-mean-square deviations (RMSD) of 1.3 Å were measured (on 1392 backbone atoms) when comparing interacting pairs of monomers in the average structure of two independent MD PduA^Sent^ (3NGK) with corresponding interacting monomers of bent BMC-H in the H. ochraceum BMC shell structure (5V74)*”.**

**Is it really necessary to write the value again in the discussion?**

Line 375: “R29N PduA: should be “N29R PduA”

**The reviewer is right. The error will be corrected, thank you.**

Line 495-496: BMC-T is not required for alpha-carboxysome minishells (Tan et al., Biomacromolecules 2021).

**This was indicated in line 496: “*while BMC-T presence was dispensable for the formation of RMM-H^Msm^ nanotubes (40), minimalist α-carboxysomes (41) or Klebsiella pneumoniae GRM2 shells (42)*”. Reference 41 corresponded to the mentioned publication from Tan et al.**

Line 497: CmcC.

**OK, it will be rectified. Thank you again.**

--------------------
