# Supplementary material for: Inferring assembly-curving trends of bacterial micro-compartment shell hexamers from crystal structure arrangements
Source: PLoS Comput Biol. 2023 Apr 5;19(4):e1011038. doi: 10.1371/journal.pcbi.1011038 (PMC10109471; doi:10.1371/journal.pcbi.1011038)
Supplement: Attachment — Submitted filename: Response to reviewers PCOMPBIOL-D-23-00208.docx [file pcbi.1011038.s017.docx]

Reviewer's Responses to Questions

**Comments to the Authors:
Please note here if the review is uploaded as an attachment.**

Reviewer #1: This is an interesting study of assembly modes of a wide range of different BMC hexamers. The authors use several different all-atom MD techniques to try and understand the spontaneous curvature of BMC assemblies, which is difficult given the computational expense. Some of the results especially for the well-studied PduA have been seen before, but in my opinion, reproduction of previous results only strengthens the reliability of the methods used and is quite useful for the community. Furthermore, this study is quite impressive in the number of different BMC hexamers which were studied including many point mutations. Overall, the conclusions are somewhat limited by the inability to measure energetic differences corresponding to different bending states as they can with different flat configurations, but it is still a good study. It should be published after addressing the following points.

Specifics:

Line 98: The authors state only the curvature of PduA has been investigated with respect to closure. I would modify that statement slightly as Mills, et al. Nat Commun 13, 3746 (2022) investigated the role of PduN (which is a pentamer) and its interaction with PduA in the context of shell closure, measuring intrinsic curvature in the interactions.

REPLY: We agree. The corresponding paragraph of the introduction has been modified to cite the reference. We also included a sentence in the discussion that mentions results from this publication (L 447).

Line 167, tri-hexamer section: My understanding is that the bending angle distributions include all 3 measured bending angles from the 3 interfaces in the tri-hexamer configuration. I am wondering if there are correlations between the 3 bending angles which were measured simultaneously for the 3 different pair interfaces in 3 hexamer configurations. In other words, does knowing the bending angle of one interface tell you what the angle of the other 2 interfaces? I assume they must be somehow correlated based on geometry. Can the authors comment on this somewhere in this section?

REPLY: this point was indeed considered in the beginning of our study. At that time, we monitored changes of bending and tilting angles for separated interfaces between hexamer pairs. Most likely because bending occurred rapidly, within the first nanosecond of the run, we did not notice about any evident correlation. That explains why data collected for the three interfaces were pooled together in the presentation of results (e.g. in Fig. 3).

As an example, the next plots present the tilting and bending angles measured for each hexamer couple with regard to other pairs (A, B and C used to name each hexamer of the tri-hexamer ensemble) for two independent MD runs with PduA*^Sent^*:

1^st^ MD:

2^nd^ MD:

Data collected for this second MD on PduA^Sent^ and a few other Arr-A cases would suggest, however, that a simultaneous evolution of bending angles occur at the beginning of the simulation:

Such data were scarce, and manifested only in some runs since. That is why we opted by not mentioning them.

However, following the suggestion of the reviewer, we added a sentence commenting this point in L199-200: “*A simultaneous evolution of angles for the three different hexamer pairs of the tri-hexamer was noticed in the beginning of some MD runs, suggesting the occurrence of coordinated movements.”*

Line 213, bi-hexamer section: This goes along with the previous point, but do you expect to see the same bending angles in a bi-hexamer as a tri-hexamer configuration? You mention cooperative effects in the manuscript and choose tri-hexamer configurations where possible (as opposed to the less computationally expensive bi-hexamer) so I am wondering if you believe it would be appropriate to assume the same bending angles would be found in a tri-hexamer configuration. Was such a hypothesis tested here or previously? You mention even on line 171 the tri-hexamer is a compromise. Can you comment on this in this section?

REPLY: In our opinion, the same angles should not be expected for bi or tri-hexamers. Breaking of planarity might be harder to achieve as the system size increases, something that could fit to the smaller angles calculated for PduA*^Sent^* tri-hexamers (27°) than those reported for hexamer pairs (34°) by Mills *et al.* Nat Commun 13, 3746 (2022). That is why, intuitively, we considered a tri-hexamer to be a better compromise, also as a means to stabilize the system, since interactions that hold couples of hexamers together were already known to be weak.

However, this point was not really tested.

Fig 4: The Potential of Mean Force is labeled as E, but this normally corresponds to an enthalpy or internal energy, whereas the authors calculated a free energy, which is a more useful quantity. I think this label should be changed from E to F or PMF to make it clear that this is a free energy calculation as opposed to just measuring energies in a simulation as in Figure S5.

REPLY: We agree. The label has been altered.

Reviewer #2: The authors did a decent job in addressing the reviewer's questions, suggestions, and concerns.

MINOR ISSUES:
L 179/180: Referring to running several short simulations versus a single long one, the authors write: "This parameter is known to impact MD trajectories more profoundly than time length (20), something that we indeed confirmed (see below)." This statement is not generally true, but it depends on whether one has an activated process (with an activation barrier), which leads to a rare event that, when it happens, is in itself fast (but the system has along dwell time in the initial minimum), or whether the process is in itself slow. The latter can be, for example, a slow phase transition or sth that is related to mass transport/diffusion over long distances, etc. In this case case, one obviously really needs long simulations. In the former case, running several short simulations can indeed be better (and is trivially parallelisable on a HPC machine).

REPLY: Certainly. We apologize for the over-simplifying commentary. We modified the corresponding paragraph to better explain our choice.

L 200 (and several places elsewhere in the text): There are different AMBER and CHARMM force field parameter sets. So it would be helpful it the authors would always specify which one exactly they refer to. Concerning the statement in L 200 that the results were reproduced with the CHARMM force field, are these data shown anywhere (in SI)? If not, this will probably have to be removed.

REPLY: We have added indications to clarify the force field used in the different sections (e.g. L 175, L 436).

With regard to CHARMM simulations, average bending and tilting angles were just mentioned in the cited paragraph of the main text. From our point of view, showing that similar qualitative results were obtained using two force fields adds credit to the study. However, in an attempt to satisfy reviewer’s demand, we added the data together with statistical value deviations as two new entries in Table S3.

We also corrected a typewriting error in L200, where bending values for CsoS1A and PduA were inadvertently inversed.

Some wording is strange here and there. One example is in L 210: "distribution of snapshot angles"? Obviously, there is no angle between snapshots, so this sentence has a logic problem. What is meant is the angle distributions calculated from the MD configurations (snapshots). Maybe the authors could carefully check the text for such things. In addition, the word "disposition" is still used at several places in the text. "Disposition" simply means sth else in English than what the authors intend to say, so perhaps correct this everywhere.

REPLY: We apologize. We have replaced the mentioned sentence and tried to improve the text. We also fully removed the “disposition” term from the text, also from legends of figures.

Reviewer #3: The authors have fully addressed my comments and made all requested changes, and I feel that the manuscript is ready for publication. I do have one additional suggestion though, that the authors include the plot of the average energies (in their response to my first "main point") in the Supporting Information of the manuscript so that readers can compare the single-configuration energies to the average energies.

REPLY: Plots presenting the averaged contribution of selected residues to the potential energy are now included in Fig. S5, in the form of a new panel C. In addition, we commented on the similarity of data obtained by the two means in the main text, L240-245.

**Have the authors made all data and (if applicable) computational code underlying the findings in their manuscript fully available?**
The [PLOS Data policy](https://journals.plos.org/ploscompbiol/s/materials-and-software-sharing) requires authors to make all data and code underlying the findings described in their manuscript fully available without restriction, with rare exception (please refer to the Data Availability Statement in the manuscript PDF file). The data and code should be provided as part of the manuscript or its supporting information, or deposited to a public repository. For example, in addition to summary statistics, the data points behind means, medians and variance measures should be available. If there are restrictions on publicly sharing data or code —e.g. participant privacy or use of data from a third party—those must be specified.

Reviewer #1: Yes

Reviewer #2: None

Reviewer #3: None

We are submitting along with the revised version a compressed file that provides the typically executed YASARA script as well all input files that would be required to reproduce our MD simulations. The contents of this .zip file are explained in the accompanying README document.

We provide also three excel file which contain raw readings of bending/tilting angles and distances extracted from individual MD snapshots, which were exploited in the generation of data summarized in the different tables of the study. Data organization inside these files is also explained in the README document.

PLOS authors have the option to publish the peer review history of their article ([what does this mean?](https://journals.plos.org/ploscompbiol/s/editorial-and-peer-review-process#loc-peer-review-history)). If published, this will include your full peer review and any attached files.


If you choose “no”, your identity will remain anonymous but your review may still be made public.


**Do you want your identity to be public for this peer review?** For information about this choice, including consent withdrawal, please see our [Privacy Policy](https://www.plos.org/privacy-policy).

Reviewer #1: No

Reviewer #2: No

Reviewer #3: No

Figure Files:

While revising your submission, please upload your figure files to the Preflight Analysis and Conversion Engine (PACE) digital diagnostic tool, https://pacev2.apexcovantage.com. PACE helps ensure that figures meet PLOS requirements. To use PACE, you must first register as a user. Then, login and navigate to the UPLOAD tab, where you will find detailed instructions on how to use the tool. If you encounter any issues or have any questions when using PACE, please email us at figures@plos.org.

Data Requirements:

Please note that, as a condition of publication, PLOS' data policy requires that you make available all data used to draw the conclusions outlined in your manuscript. Data must be deposited in an appropriate repository, included within the body of the manuscript, or uploaded as supporting information. This includes all numerical values that were used to generate graphs, histograms etc.. For an example in PLOS Biology see here: http://www.plosbiology.org/article/info%3Adoi%2F10.1371%2Fjournal.pbio.1001908#s5.

Reproducibility:

To enhance the reproducibility of your results, we recommend that you deposit your laboratory protocols in protocols.io, where a protocol can be assigned its own identifier (DOI) such that it can be cited independently in the future. Additionally, PLOS ONE offers an option to publish peer-reviewed clinical study protocols. Read more information on sharing protocols at <https://plos.org/protocols?utm_medium=editorial-email&utm_source=authorletters&utm_campaign=protocols>

References:

Review your reference list to ensure that it is complete and correct. If you have cited papers that have been retracted, please include the rationale for doing so in the manuscript text, or remove these references and replace them with relevant current references. Any changes to the reference list should be mentioned in the rebuttal letter that accompanies your revised manuscript.

*If you need to cite a retracted article, indicate the article’s retracted status in the References list and also include a citation and full reference for the retraction notice.*

*In compliance with data protection regulations, you may request that we remove your personal registration details at any time.* [*(Remove my information/details)*](https://www.editorialmanager.com/pcompbiol/login.asp?a=r)*. Please contact the publication office if you have any questions.*
